# Supplementary material for: pH-dependent and dynamic interactions of cystatin C with heparan sulfate
Source: Commun Biol. 2021 Feb 12;4:198. doi: 10.1038/s42003-021-01737-7 (PMC7881039; doi:10.1038/s42003-021-01737-7)
Supplement: Supplementary file 2 — Supplementary Information [file 42003_2021_1737_MOESM2_ESM.pdf]

Supplemental Figure 1

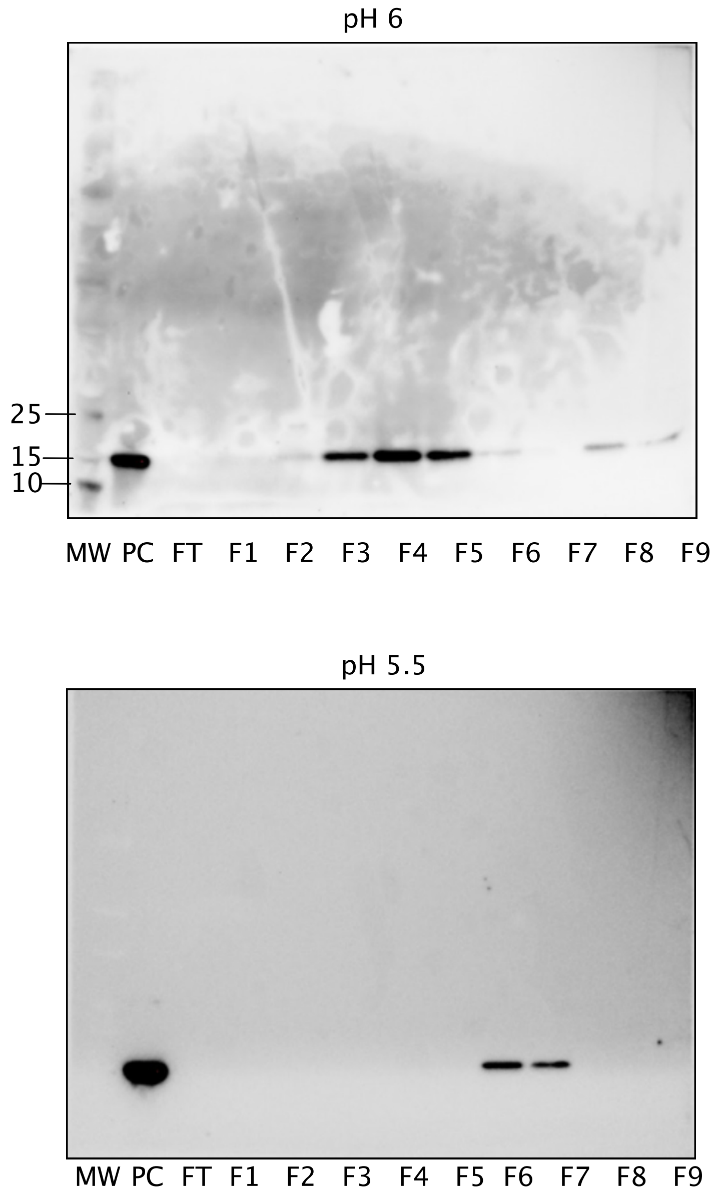

Supplemental figure 1. Western blot analysis of heparin Sepharose chromatography fractions of 293 cells expressed murine Cst-3. Full blot for images presented in Fig. 1b.

Supplemental Figure 2

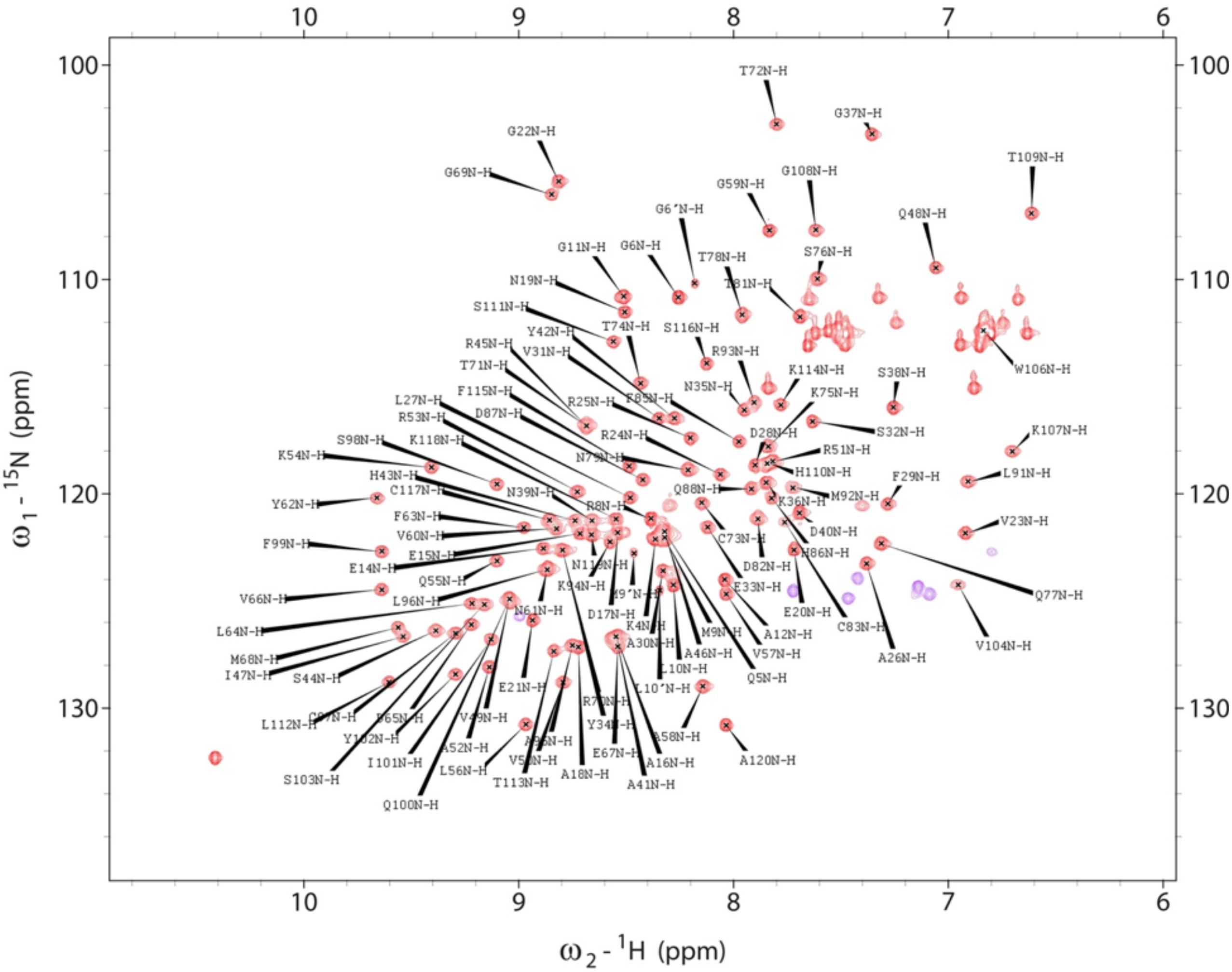

Supplemental figure 2. Backbone assignment in ^{1}H-^{15}N HSQC spectrum of Cst-3.

Supplemental Figure 3

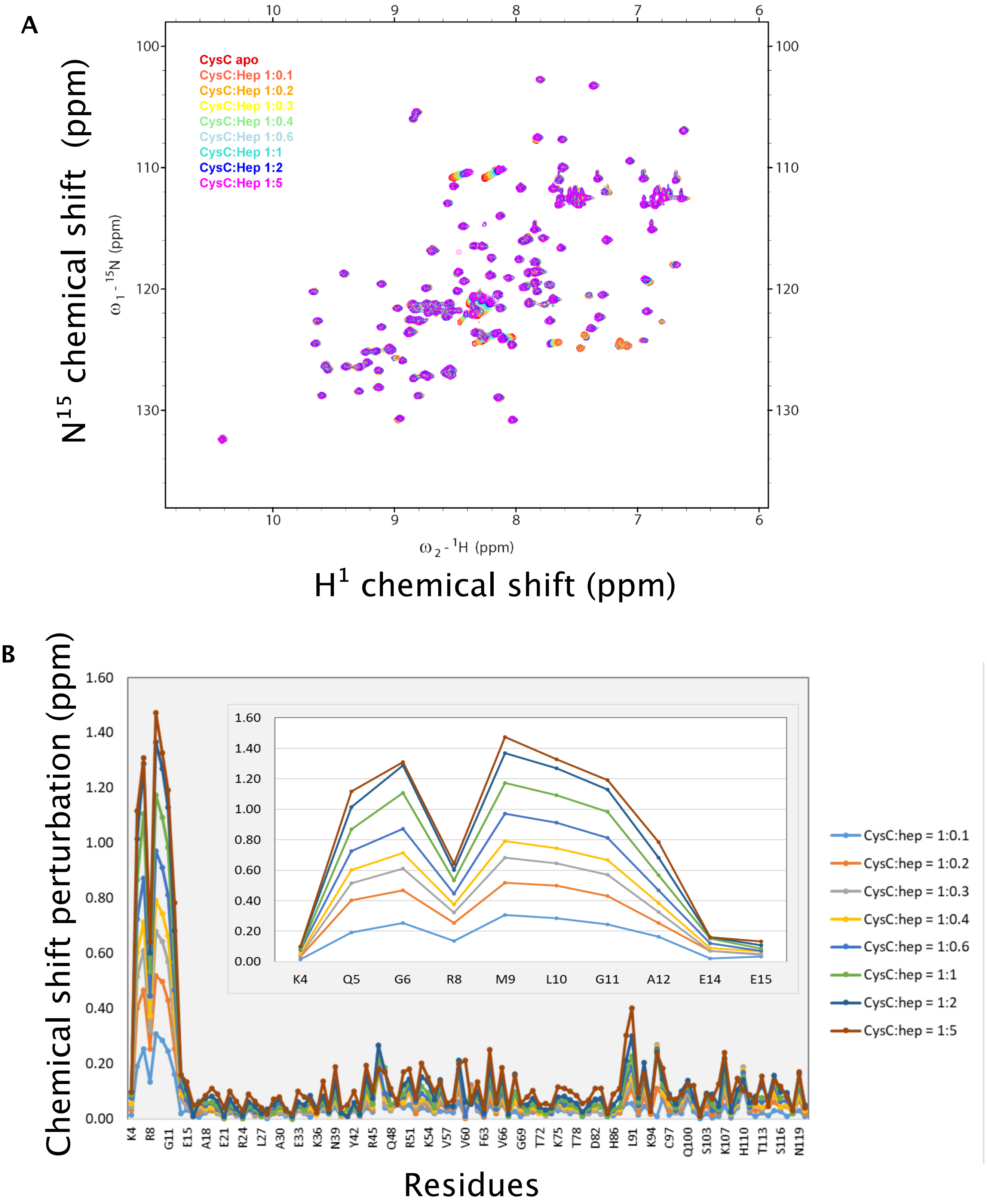

Supplemental figure 3. NMR titration analysis of the interaction between Cst-3 and heparin at pH 5.5. (A) HSQC spectra showed that several residues went through significant chemical shift upon heparin-titration. (B) HSQC peaks of each residue upon heparin-titration revealed that the largest CSP was near the N terminus.

Supplemental Figure 4

```
                       4   8                                          51
murine_Cst-3        ATPKQGPRMLGAPEEADANEEGVRRALDFAVSEYNKGSNDAYHSRAIQVVRARKQLVAGV  60
human_Cst-3         SSPGKPPRLVGGPMDASVEEEGVRRALDFAVGEYNKASNDMYHSRALQVVRARKQIVAGV  60
                    ::* : **::*.* :*..:************.****.*** *****:********:****

                             70                  90 93-94
murine_Cst-3        NYFLDVEMGRTTCTKSQTNLTDCPFHDQPHLMRKALCSFQIYSVPWKGTHSLTKFSCKNA  120
human_Cst-3         NYFLDVELGRTTCTKTQPNLDNCPFHDQPHLKRKAFCSFQIYAVPWQGTMTLSKSTCQDA  120
                    *******:*******:* ** :********* ***:******:***:** :*:* :*::*
```

Supplemental figure 4. Sequnence aligment of murine (21-140) and human Cst-3(27-146). The signal peptides were not included in the alignment. Identified HS-binding residues are boxed.

Supplemental Figure 5

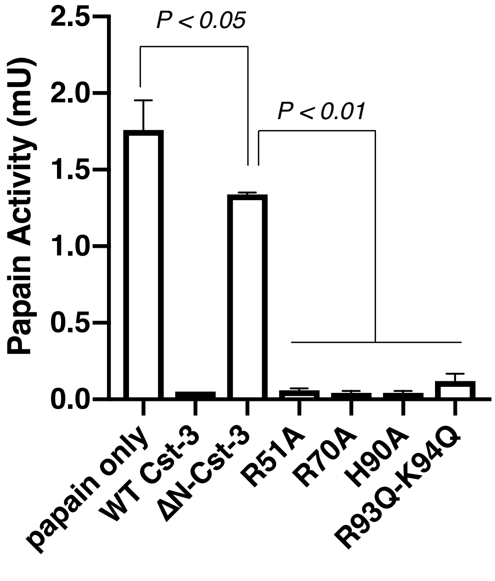

Supplemental figure 5. Inhibitory activity of various Cst-3 mutants towards papain. The enzymatic activity of papain was determined by using a colorimetric peptide substrate in the presence of wild-type Cst-3, ΔN-Cst-3, R51A, R70A, H90A and R93Q-K94Q .
