# Supplementary material for: pH-dependent and dynamic interactions of cystatin C with heparan sulfate
Source: Commun Biol. 2021 Feb 12;4:198. doi: 10.1038/s42003-021-01737-7 (PMC7881039; doi:10.1038/s42003-021-01737-7)
Supplement: Supplementary file 3 — Description of Additional Supplementary Files [file 42003_2021_1737_MOESM3_ESM.pdf]

Description of Additional Supplementary Items

File Name: Supplementary Data 1-2

Description: Source data for the main figures
