# Supplementary material for: pH-dependent and dynamic interactions of cystatin C with heparan sulfate
Source: Commun Biol. 2021 Feb 12;4:198. doi: 10.1038/s42003-021-01737-7 (PMC7881039; doi:10.1038/s42003-021-01737-7)
Supplement: Supplementary file 4 — Supplementary Data 1 [file 42003_2021_1737_MOESM4_ESM.pdf]

ATF 1
30 41
Type=GenePix Results 3
DateTime=2019/02/11 17:57:43
Settings=
GalFile=
PixelSize=5
Wavelengths=488
ImageFiles=C:\Users\sop_jliulab.inst\Desktop\image\2019-02-11-10250977-Cysc-Biotin x Cysc-488Avidin-500-1
NormalizationMethod=None
NormalizationFactors=1
JpegImage=
StdDev=Type 1
RatioFormulations=W4/W1 (488/#1)
FeatureType=Circular
Barcode=
BackgroundSubtraction=LocalFeature
ImageOrigin=0, 0
JpegOrigin=3265, 10250
Creator=GenePix Pro 7.2.29.002
Scanner=GenePix 4300 [141086]
FocusPosition=0
Temperature=0
LinesAveraged=1
Comment=
PMTGain=500
ScanPower=100
LaserPower=2.12
Filters=Standard Blue
ScanRegion=353,970,4001,13353
ScanArea=1
Supplier=

Flags	Norm	Autofla	Block	Column	Row	X	Y	Dia.	F488 Media	F488 Mean	F488 SD
0	0	0	1	1	1	3425	10380	70	175	204	109
0	0	0	1	2	1	3810	10390	70	431	420	129
0	0	0	1	3	1	4190	10400	75	363	356	149
0	0	0	1	4	1	4570	10400	75	421	411	123
0	0	0	1	5	1	4945	10400	75	354	356	151
0	0	0	1	6	1	5320	10385	70	418	430	123
0	0	0	1	1	2	3435	10760	70	390	388	152
0	0	0	1	2	2	3825	10775	80	325	342	211
0	0	0	1	3	2	4175	10785	45	266	278	145
0	0	0	1	4	2	4570	10795	85	314	305	115
0	0	0	1	5	2	4930	10790	75	286	291	118
0	0	0	1	6	2	5305	10765	75	383	387	96
0	0	0	1	1	3	3440	11135	70	416	397	124
0	0	0	1	2	3	3840	11140	85	241	249	121
0	0	0	1	3	3	4225	11155	95	281	287	77

0	0	0	1	4	3	4550	11175	95	293	298	81
0	0	0	1	5	3	4905	11150	90	275	279	92
0	0	0	1	6	3	5300	11140	75	415	479	510
0	0	0	1	1	4	3440	11520	70	383	368	99
0	0	0	1	2	4	3840	11510	80	295	296	107
0	0	0	1	3	4	4220	11495	90	258	257	95
0	0	0	1	4	4	4550	11480	90	210	224	107
0	0	0	1	5	4	4905	11505	90	267	275	94
0	0	0	1	6	4	5295	11520	75	383	388	116
0	0	0	1	1	5	3435	11900	70	384	386	111
0	0	0	1	2	5	3820	11890	80	197	216	160
0	0	0	1	3	5	4200	11875	80	314	326	118
0	0	0	1	4	5	4565	11870	85	298	309	106
0	0	0	1	5	5	4925	11885	85	353	359	97
0	0	0	1	6	5	5305	11905	75	380	384	114
0	0	0	1	1	6	3420	12280	75	472	489	212
0	0	0	1	2	6	3810	12270	70	387	388	106
0	0	0	1	3	6	4190	12260	70	398	396	121
0	0	0	1	4	6	4570	12255	75	307	302	149
0	0	0	1	5	6	4945	12265	75	241	267	165
0	0	0	1	6	6	5320	12280	70	511	596	1064
0	0	0	2	1	1	3430	14285	55	304	305	119
0	0	0	2	2	1	3815	14300	50	409	410	125
0	0	0	2	3	1	4195	14310	50	389	409	138
0	0	0	2	4	1	4570	14310	55	506	531	183
0	0	0	2	5	1	4945	14310	55	599	644	263
0	0	0	2	6	1	5320	14290	55	481	585	485
0	0	0	2	1	2	3450	14660	50	321	339	110
0	0	0	2	2	2	3830	14685	55	426	462	176
0	0	0	2	3	2	4210	14695	65	429	452	188
0	0	0	2	4	2	4565	14705	65	524	600	367
0	0	0	2	5	2	4925	14690	60	427	428	138
0	0	0	2	6	2	5300	14670	55	452	475	138
0	0	0	2	1	3	3450	15045	50	384	426	194
0	0	0	2	2	3	3845	15055	60	495	504	199
0	0	0	2	3	3	4225	15065	75	438	446	137
0	0	0	2	4	3	4550	15080	80	515	527	155
0	0	0	2	5	3	4910	15060	70	410	414	158
0	0	0	2	6	3	5295	15045	55	620	641	149
0	0	0	2	1	4	3450	15415	50	571	608	345
0	0	0	2	2	4	3840	15405	55	532	561	203
0	0	0	2	3	4	4220	15390	65	501	554	242
0	0	0	2	4	4	4555	15380	75	458	472	120
0	0	0	2	5	4	4915	15405	65	526	536	144
0	0	0	2	6	4	5295	15425	55	629	639	169
0	0	0	2	1	5	3445	15800	50	576	662	398
0	0	0	2	2	5	3830	15780	55	623	694	421
0	0	0	2	3	5	4210	15770	65	537	528	173

0	0	0	2	4	5	4565	15765	65	586	635	220
0	0	0	2	5	5	4925	15780	60	545	560	219
0	0	0	2	6	5	5300	15800	55	604	609	176
-50	0	0	2	1	6	3470	16105	70	47	50	18
0	0	0	2	2	6	3815	16160	50	660	664	193
0	0	0	2	3	6	4195	16155	50	502	538	216
0	0	0	2	4	6	4575	16145	55	615	658	233
0	0	0	2	5	6	4950	16150	55	615	696	228
0	0	0	2	6	6	5320	16170	55	691	684	199
0	0	0	3	1	1	3425	18180	65	100	217	939
0	0	0	3	2	1	3805	18190	75	104	109	46
0	0	0	3	3	1	4190	18200	70	116	121	57
0	0	0	3	4	1	4570	18205	65	122	124	46
0	0	0	3	5	1	4940	18200	65	106	113	44
0	0	0	3	6	1	5315	18190	65	111	116	46
0	0	0	3	1	2	3440	18555	65	108	110	37
0	0	0	3	2	2	3825	18575	70	115	121	46
0	0	0	3	3	2	4205	18585	80	108	110	45
0	0	0	3	4	2	4560	18595	80	118	122	45
0	0	0	3	5	2	4925	18585	75	111	121	53
0	0	0	3	6	2	5300	18560	75	101	107	47
0	0	0	3	1	3	3440	18940	65	105	116	58
0	0	0	3	2	3	3840	18945	75	104	133	248
0	0	0	3	3	3	4220	18960	90	105	110	49
0	0	0	3	4	3	4550	18980	90	99	114	99
0	0	0	3	5	3	4910	18950	85	104	109	45
0	0	0	3	6	3	5295	18945	70	98	104	48
0	0	0	3	1	4	3445	19315	70	117	119	51
0	0	0	3	2	4	3835	19310	75	101	137	231
0	0	0	3	3	4	4215	19295	90	113	119	51
0	0	0	3	4	4	4550	19285	95	105	114	87
0	0	0	3	5	4	4910	19300	80	101	107	46
0	0	0	3	6	4	5295	19320	65	119	129	72
0	0	0	3	1	5	3435	19700	70	93	97	42
0	0	0	3	2	5	3820	19685	70	98	100	41
0	0	0	3	3	5	4195	19675	75	98	106	51
0	0	0	3	4	5	4560	19670	80	95	101	45
0	0	0	3	5	5	4920	19685	100	78	86	42
0	0	0	3	6	5	5300	19705	65	114	120	43
0	0	0	3	1	6	3425	20080	65	136	143	58
0	0	0	3	2	6	3805	20065	65	122	187	474
0	0	0	3	3	6	4190	20065	65	99	105	42
0	0	0	3	4	6	4570	20065	75	118	127	76
0	0	0	3	5	6	4945	20065	65	116	124	58
0	0	0	3	6	6	5320	20080	65	138	151	78
0	0	0	4	1	1	3420	22080	85	5205	5059	4680
0	0	0	4	2	1	3800	22095	80	7366	5604	4845
0	0	0	4	3	1	4185	22100	80	8020	5717	4809

0	0	0	4	4	1	4565	22105	85	7587	5765	4640
0	0	0	4	5	1	4940	22100	85	13472	16997	18266
0	0	0	4	6	1	5315	22085	80	7673	5594	4668
0	0	0	4	1	2	3435	22455	80	6181	5430	4796
0	0	0	4	2	2	3820	22475	90	7250	5372	4964
0	0	0	4	3	2	4195	22485	80	8034	7344	3286
0	0	0	4	4	2	4560	22490	75	8045	8196	1033
0	0	0	4	5	2	4925	22480	80	7789	6901	3529
0	0	0	4	6	2	5305	22460	80	8082	5862	4458
0	0	0	4	1	3	3435	22835	80	8204	5790	5126
0	0	0	4	2	3	3830	22845	85	8001	7013	3918
0	0	0	4	3	3	4215	22855	90	8316	8700	5209
0	0	0	4	4	3	4545	22870	90	8428	8589	1428
0	0	0	4	5	3	4910	22850	80	8351	8691	1618
0	0	0	4	6	3	5295	22840	85	8811	6502	4773
0	0	0	4	1	4	3435	23215	75	9063	7405	4511
0	0	0	4	2	4	3830	23210	90	8157	6166	4856
0	0	0	4	3	4	4210	23195	85	8402	8743	1956
0	0	0	4	4	4	4550	23185	90	8918	9127	1768
0	0	0	4	5	4	4915	23205	80	9024	9195	2276
0	0	0	4	6	4	5295	23220	85	9410	7124	5610
0	0	0	4	1	5	3430	23605	75	9712	8146	6019
0	0	0	4	2	5	3820	23590	90	8366	6027	5434
0	0	0	4	3	5	4195	23575	90	9058	6959	5222
0	0	0	4	4	5	4565	23575	75	9537	9860	1671
0	0	0	4	5	5	4930	23590	75	9833	9906	2515
0	0	0	4	6	5	5305	23605	85	9823	7243	5612
0	0	0	4	1	6	3420	23975	80	6703	6247	5769
0	0	0	4	2	6	3805	23970	75	11288	9167	5640
0	0	0	4	3	6	4190	23965	85	9575	7013	6120
0	0	0	4	4	6	4565	23960	90	1412	5866	5949
0	0	0	4	5	6	4950	23960	80	9046	6790	5503
0	0	0	4	6	6	5315	23975	85	9260	6644	5702
0	0	0	5	1	1	3420	25975	75	8444	7117	5476
0	0	0	5	2	1	3805	25990	75	8417	7736	5196
0	0	0	5	3	1	4185	25995	75	7240	6640	4192
0	0	0	5	4	1	4570	26000	70	8060	7465	4216
0	0	0	5	5	1	4945	25995	85	6687	5948	5140
0	0	0	5	6	1	5315	25985	80	6305	5407	5038
0	0	0	5	1	2	3435	26355	75	7627	6692	4660
0	0	0	5	2	2	3820	26370	70	8445	8219	3549
0	0	0	5	3	2	4200	26380	75	7546	7693	2364
0	0	0	5	4	2	4560	26390	80	7028	7182	3062
0	0	0	5	5	2	4925	26375	75	8003	8468	3012
0	0	0	5	6	2	5305	26355	80	7249	6394	5293
0	0	0	5	1	3	3440	26735	75	7233	6194	4137
0	0	0	5	2	3	3835	26745	75	7162	7505	2747
0	0	0	5	3	3	4215	26755	85	6431	6973	2386

0	0	0	5	4	3	4550	26770	95	6863	7244	1532
0	0	0	5	5	3	4910	26750	85	7616	7847	2550
0	0	0	5	6	3	5300	26735	70	9278	8321	4359
0	0	0	5	1	4	3440	27120	70	7331	6333	4285
0	0	0	5	2	4	3830	27110	75	7389	7795	3016
0	0	0	5	3	4	4215	27100	85	6599	6915	2559
0	0	0	5	4	4	4550	27080	90	6834	7057	2074
0	0	0	5	5	4	4915	27105	85	7212	7228	2653
0	0	0	5	6	4	5300	27115	70	9103	8179	4324
0	0	0	5	1	5	3435	27505	65	7628	7647	3612
0	0	0	5	2	5	3820	27490	70	7198	6952	3263
0	0	0	5	3	5	4195	27480	75	6984	7370	2844
0	0	0	5	4	5	4565	27475	80	7024	7118	3282
0	0	0	5	5	5	4925	27485	70	8371	8289	2855
0	0	0	5	6	5	5305	27505	75	9263	8431	4935
0	0	0	5	1	6	3420	27880	70	5516	4454	3372
0	0	0	5	2	6	3805	27865	75	6662	5671	4137
0	0	0	5	3	6	4185	27860	75	8115	7446	5170
0	0	0	5	4	6	4570	27865	75	8743	8037	4790
0	0	0	5	5	6	4950	27865	80	7570	6425	5587
0	0	0	5	6	6	5320	27880	75	9323	8280	5394
0	0	0	6	1	1	3420	29885	60	3915	3444	1799
0	0	0	6	2	1	3805	29900	60	3892	3591	1804
0	0	0	6	3	1	4185	29905	60	5966	5568	2643
0	0	0	6	4	1	4560	29910	70	5688	4660	3155
0	0	0	6	5	1	4940	29905	70	5385	4469	3029
0	0	0	6	6	1	5310	29890	75	7034	5508	4016
0	0	0	6	1	2	3435	30260	60	3774	3353	1665
0	0	0	6	2	2	3820	30280	65	3841	3830	886
0	0	0	6	3	2	4200	30300	65	4100	4479	1341
0	0	0	6	4	2	4560	30300	70	5354	5686	1617
0	0	0	6	5	2	4920	30290	70	6313	6496	3170
0	0	0	6	6	2	5300	30270	65	7857	8277	2974
0	0	0	6	1	3	3440	30645	70	3480	2709	2077
0	0	0	6	2	3	3840	30655	90	1776	1978	1831
0	0	0	6	3	3	4215	30665	80	3223	3282	1173
0	0	0	6	4	3	4555	30680	85	5334	5501	1170
0	0	0	6	5	3	4905	30655	75	6926	7397	1902
0	0	0	6	6	3	5290	30645	70	9894	9737	8705
0	0	0	6	1	4	3435	31020	60	3975	3615	1744
0	0	0	6	2	4	3835	31010	60	3680	3710	708
0	0	0	6	3	4	4210	30990	75	3143	3254	714
0	0	0	6	4	4	4550	30985	90	5888	5729	2280
0	0	0	6	5	4	4910	31005	75	7530	7918	2197
0	0	0	6	6	4	5290	31020	70	7382	6299	4208
0	0	0	6	1	5	3435	31400	60	4017	3415	1805
0	0	0	6	2	5	3820	31390	65	3975	3916	1229
0	0	0	6	3	5	4195	31380	70	4218	4006	1576

0	0	0	6	4	5	4560	31375	70	6769	7091	2063
0	0	0	6	5	5	4920	31385	70	7028	7126	3472
0	0	0	6	6	5	5295	31405	80	3703	3856	3754
0	0	0	6	1	6	3420	31780	60	3798	3416	1969
0	0	0	6	2	6	3805	31765	60	3881	3290	1687
0	0	0	6	3	6	4190	31755	65	4087	3703	1808
0	0	0	6	4	6	4565	31760	75	7244	5800	4113
0	0	0	6	5	6	4945	31760	75	6701	5544	3827
0	0	0	6	6	6	5315	31775	65	6103	5932	2983
0	0	0	7	1	1	3415	33780	70	4401	4161	1623
0	0	0	7	2	1	3795	33790	70	7456	7269	2223
0	0	0	7	3	1	4180	33800	70	8265	8122	2614
0	0	0	7	4	1	4560	33800	70	9235	9971	4337
0	0	0	7	5	1	4940	33800	75	9696	9137	3339
0	0	0	7	6	1	5315	33785	70	9847	9938	2643
0	0	0	7	1	2	3425	34155	70	4421	4079	1390
0	0	0	7	2	2	3815	34170	80	4279	3926	1612
0	0	0	7	3	2	4190	34185	85	7671	7936	2001
0	0	0	7	4	2	4555	34190	80	7491	7743	1563
0	0	0	7	5	2	4920	34180	85	8785	8779	2501
0	0	0	7	6	2	5300	34160	80	9917	8696	4729
0	0	0	7	1	3	3430	34535	70	4288	4341	1388
0	0	0	7	2	3	3825	34545	80	4847	4870	1428
0	0	0	7	3	3	4210	34555	95	5179	5303	1136
0	0	0	7	4	3	4545	34570	100	6921	7045	1998
0	0	0	7	5	3	4905	34550	90	7602	7904	2154
0	0	0	7	6	3	5295	34540	75	10365	10722	2149
0	0	0	7	1	4	3430	34920	70	4410	4244	1495
0	0	0	7	2	4	3825	34910	80	5738	5641	1827
0	0	0	7	3	4	4205	34895	95	4063	4114	765
0	0	0	7	4	4	4545	34885	100	6430	6322	1641
0	0	0	7	5	4	4905	34905	85	8321	8654	1581
0	0	0	7	6	4	5295	34920	75	10869	11169	2381
0	0	0	7	1	5	3425	35305	70	5101	4800	1895
0	0	0	7	2	5	3815	35290	75	6432	6443	1530
0	0	0	7	3	5	4195	35280	80	5162	4972	1458
0	0	0	7	4	5	4555	35275	85	5308	5305	1047
0	0	0	7	5	5	4925	35285	80	10270	10476	3055
0	0	0	7	6	5	5300	35305	85	10440	8829	5132
0	0	0	7	1	6	3415	35680	70	5770	5166	2110
0	0	0	7	2	6	3800	35670	70	5471	5385	2063
0	0	0	7	3	6	4175	35665	65	6764	6669	1312
0	0	0	7	4	6	4565	35665	70	5360	5205	1391
0	0	0	7	5	6	4940	35670	70	9705	9498	2673
0	0	0	7	6	6	5320	35680	85	10627	8494	5475
0	0	0	8	1	1	3420	37680	85	5726	4314	3248
0	0	0	8	2	1	3800	37690	80	5655	4311	2991
0	0	0	8	3	1	4185	37695	75	5596	5257	2014

0	0	0	8	4	1	4565	37700	70	5872	5726	1476
0	0	0	8	5	1	4945	37695	75	6035	5835	1693
0	0	0	8	6	1	5315	37685	85	9824	7965	5360
0	0	0	8	1	2	3430	38055	80	5471	4403	3250
0	0	0	8	2	2	3815	38070	75	5801	5964	1424
0	0	0	8	3	2	4195	38085	80	4908	4876	1302
0	0	0	8	4	2	4560	38095	85	4634	4804	894
0	0	0	8	5	2	4925	38080	80	5311	5249	1893
0	0	0	8	6	2	5300	38060	85	9518	7891	4694
0	0	0	8	1	3	3435	38435	70	5604	5187	1896
0	0	0	8	2	3	3830	38445	80	4996	4777	1679
0	0	0	8	3	3	4215	38455	95	4243	4399	975
0	0	0	8	4	3	4545	38475	100	4093	4146	992
0	0	0	8	5	3	4905	38450	90	4401	4364	1419
0	0	0	8	6	3	5295	38445	85	9375	8114	4537
0	0	0	8	1	4	3435	38820	85	5127	4160	2899
0	0	0	8	2	4	3825	38810	75	4929	5031	950
0	0	0	8	3	4	4210	38800	95	4694	4784	1065
0	0	0	8	4	4	4545	38785	95	4262	4402	757
0	0	0	8	5	4	4910	38805	85	4598	4775	840
0	0	0	8	6	4	5300	38820	75	10245	10176	2687
0	0	0	8	1	5	3430	39205	85	5165	4117	2956
0	0	0	8	2	5	3815	39195	70	5778	5926	1187
0	0	0	8	3	5	4195	39180	80	5911	5886	2009
0	0	0	8	4	5	4560	39180	80	4808	4950	1027
0	0	0	8	5	5	4925	39190	80	5344	5145	1613
0	0	0	8	6	5	5300	39210	80	6912	5624	3328
0	0	0	8	1	6	3420	39580	80	5284	4051	2952
0	0	0	8	2	6	3805	39570	75	5355	4762	2107
0	0	0	8	3	6	4185	39565	70	6032	5616	2223
0	0	0	8	4	6	4565	39565	70	6626	6144	2192
0	0	0	8	5	6	4945	39570	75	6155	5803	1855
0	0	0	8	6	6	5315	39580	85	6388	4913	3282
0	0	0	9	1	1	3430	41580	60	1312	1303	554
0	0	0	9	2	1	3800	41595	60	1374	1324	505
0	0	0	9	3	1	4200	41600	60	1431	1430	500
0	0	0	9	4	1	4565	41600	60	1581	1654	395
0	0	0	9	5	1	4950	41600	65	1568	1545	447
0	0	0	9	6	1	5325	41590	60	1478	1413	533
0	0	0	9	1	2	3445	41950	60	1387	1376	540
0	0	0	9	2	2	3825	41970	65	1389	1434	365
0	0	0	9	3	2	4205	41980	70	1145	1266	641
0	0	0	9	4	2	4570	41990	70	1321	1364	373
0	0	0	9	5	2	4920	41985	70	1374	1395	404
-50	0	0	9	6	2	5290	41985	70	42	48	25
0	0	0	9	1	3	3440	42340	60	1193	1199	460
0	0	0	9	2	3	3835	42345	65	1117	1142	261
0	0	0	9	3	3	4215	42375	85	1122	1160	360

0	0	0	9	4	3	4560	42370	90	1110	1130	299
0	0	0	9	5	3	4915	42345	80	1492	1486	440
0	0	0	9	6	3	5300	42345	60	1636	1766	1692
0	0	0	9	1	4	3440	42715	60	1189	1195	308
0	0	0	9	2	4	3830	42705	65	1053	1079	262
0	0	0	9	3	4	4220	42690	80	1021	1029	303
0	0	0	9	4	4	4540	42675	85	1001	1043	246
0	0	0	9	5	4	4905	42705	75	1305	1378	380
0	0	0	9	6	4	5300	42710	65	1454	1409	425
0	0	0	9	1	5	3430	43090	55	1017	1025	252
0	0	0	9	2	5	3825	43080	60	1141	1172	304
0	0	0	9	3	5	4190	43070	70	1272	1305	449
0	0	0	9	4	5	4565	43070	75	1356	1397	359
0	0	0	9	5	5	4925	43085	70	1648	1670	832
0	0	0	9	6	5	5310	43095	65	1424	1386	419
0	0	0	9	1	6	3415	43470	60	1179	1165	445
0	0	0	9	2	6	3805	43465	60	1191	1178	361
0	0	0	9	3	6	4210	43465	60	1167	1175	435
0	0	0	9	4	6	4580	43455	60	1652	1649	436
0	0	0	9	5	6	4950	43465	60	1477	1507	401
0	0	0	9	6	6	5325	43480	60	1384	1330	442
0	0	0	10	1	1	3410	45480	80	6120	4385	3244
0	0	0	10	2	1	3790	45490	75	6754	5990	2485
0	0	0	10	3	1	4170	45505	75	6838	5884	2675
0	0	0	10	4	1	4560	45510	85	5903	4558	2982
0	0	0	10	5	1	4935	45505	70	6265	5807	2125
0	0	0	10	6	1	5305	45490	80	6564	4933	3734
0	0	0	10	1	2	3420	45865	90	2298	3570	3440
0	0	0	10	2	2	3815	45870	85	6010	4867	2738
0	0	0	10	3	2	4195	45875	80	6008	5847	1737
0	0	0	10	4	2	4550	45895	80	5553	5755	1108
0	0	0	10	5	2	4915	45890	85	5502	5107	2002
0	0	0	10	6	2	5305	45865	75	6865	6754	1516
0	0	0	10	1	3	3425	46255	90	3547	3440	3193
0	0	0	10	2	3	3815	46245	80	5721	5122	2336
0	0	0	10	3	3	4200	46260	90	5008	5001	1219
0	0	0	10	4	3	4525	46290	100	5474	6106	5186
0	0	0	10	5	3	4895	46235	85	5347	5497	932
0	0	0	10	6	3	5290	46245	70	6412	6276	1383
0	0	0	10	1	4	3430	46630	85	6135	4600	3323
0	0	0	10	2	4	3820	46615	75	5763	5872	1010
0	0	0	10	3	4	4200	46590	90	5433	5620	2852
0	0	0	10	4	4	4535	46585	95	5338	5602	1539
0	0	0	10	5	4	4910	46605	85	5489	5849	3393
0	0	0	10	6	4	5285	46620	75	6581	6214	1709
0	0	0	10	1	5	3425	47005	90	1255	3188	3140
0	0	0	10	2	5	3810	46995	90	5205	3612	2884
0	0	0	10	3	5	4195	46995	80	5695	5381	2029

0	0	0	10	4	5	4550	46985	80	6010	6203	1268
0	0	0	10	5	5	4920	46985	75	5618	5837	993
0	0	0	10	6	5	5290	47005	80	5949	4576	2936
0	0	0	10	1	6	3415	47390	85	6011	4243	3376
0	0	0	10	2	6	3790	47375	80	6144	4528	3887
0	0	0	10	3	6	4185	47365	75	6423	6427	5858
0	0	0	10	4	6	4565	47365	75	6613	5972	2389
0	0	0	10	5	6	4940	47370	75	6340	5784	2046
0	0	0	10	6	6	5305	47380	75	6555	5723	2391
0	0	0	11	1	1	3400	49380	75	4451	4131	1562
0	0	0	11	2	1	3785	49395	80	4364	3651	2163
0	0	0	11	3	1	4170	49400	70	4131	4046	1057
0	0	0	11	4	1	4550	49405	70	4185	4096	1052
0	0	0	11	5	1	4930	49400	70	4606	4691	1027
0	0	0	11	6	1	5300	49385	75	4452	4275	1082
0	0	0	11	1	2	3415	49750	70	4156	3911	1126
0	0	0	11	2	2	3805	49770	80	3324	3136	1400
0	0	0	11	3	2	4175	49790	80	3116	3220	968
0	0	0	11	4	2	4550	49805	85	2762	3016	792
0	0	0	11	5	2	4925	49785	80	3190	3173	1102
0	0	0	11	6	2	5290	49770	75	4321	4420	1547
0	0	0	11	1	3	3420	50150	70	3955	3862	1108
0	0	0	11	2	3	3815	50150	80	3270	3246	1038
0	0	0	11	3	3	4210	50160	95	2544	2805	836
0	0	0	11	4	3	4520	50185	100	2544	2787	2036
0	0	0	11	5	3	4895	50155	95	2694	2896	1001
0	0	0	11	6	3	5285	50160	75	3665	3752	1265
0	0	0	11	1	4	3420	50525	75	4284	4985	5762
0	0	0	11	2	4	3820	50520	80	3095	3039	1067
0	0	0	11	3	4	4195	50495	95	2442	2640	799
0	0	0	11	4	4	4535	50490	95	2359	2498	636
0	0	0	11	5	4	4905	50520	90	2514	2915	3847
0	0	0	11	6	4	5285	50525	75	3685	3637	709
0	0	0	11	1	5	3420	50905	70	3385	3179	1156
0	0	0	11	2	5	3805	50900	75	3440	3483	538
0	0	0	11	3	5	4190	50880	85	2785	2799	900
0	0	0	11	4	5	4535	50880	80	2399	2528	697
0	0	0	11	5	5	4910	50895	85	3037	3128	1317
0	0	0	11	6	5	5295	50905	75	3716	3693	743
0	0	0	11	1	6	3400	51285	70	3379	3269	1449
0	0	0	11	2	6	3795	51280	80	3565	2831	1761
0	0	0	11	3	6	4165	51270	70	3531	3339	1196
0	0	0	11	4	6	4565	51270	75	3998	5576	7769
0	0	0	11	5	6	4935	51270	65	4232	4371	1007
0	0	0	11	6	6	5310	51285	70	3996	3835	1113
0	0	0	12	1	1	3395	53290	55	439	439	119
0	0	0	12	2	1	3770	53310	55	444	467	170
0	0	0	12	3	1	4160	53310	55	519	530	169

0	0	0	12	4	1	4545	53315	55	736	734	178
0	0	0	12	5	1	4910	53315	60	1152	1223	820
0	0	0	12	6	1	5285	53285	50	545	546	192
0	0	0	12	1	2	3415	53665	55	360	407	172
0	0	0	12	2	2	3795	53685	65	457	474	153
0	0	0	12	3	2	4170	53690	65	421	433	133
0	0	0	12	4	2	4540	53710	70	391	426	213
0	0	0	12	5	2	4895	53690	65	384	398	150
0	0	0	12	6	2	5270	53660	60	493	471	177
0	0	0	12	1	3	3415	54045	60	436	410	201
0	0	0	12	2	3	3800	54065	60	390	402	150
0	0	0	12	3	3	4190	54065	75	323	338	131
0	0	0	12	4	3	4520	54070	85	385	409	144
0	0	0	12	5	3	4880	54065	70	463	462	146
0	0	0	12	6	3	5265	54050	60	503	505	226
0	0	0	12	1	4	3415	54420	55	448	448	121
0	0	0	12	2	4	3805	54415	65	416	449	182
0	0	0	12	3	4	4185	54390	80	344	340	163
0	0	0	12	4	4	4520	54385	85	408	490	394
0	0	0	12	5	4	4880	54405	75	418	448	162
0	0	0	12	6	4	5260	54435	60	708	719	351
0	0	0	12	1	5	3410	54815	60	273	256	125
0	0	0	12	2	5	3795	54790	60	446	439	124
0	0	0	12	3	5	4180	54775	65	325	335	107
0	0	0	12	4	5	4525	54760	65	406	426	137
0	0	0	12	5	5	4895	54785	65	355	355	128
0	0	0	12	6	5	5280	54800	60	469	482	202
0	0	0	12	1	6	3380	55165	60	443	591	779
0	0	0	12	2	6	3780	55170	60	344	349	192
0	0	0	12	3	6	4160	55165	55	426	465	152
0	0	0	12	4	6	4540	55160	55	486	477	120
0	0	0	12	5	6	4915	55165	55	373	388	121
0	0	0	12	6	6	5285	55180	55	357	366	98
0	0	0	13	1	1	3385	57180	60	956	940	252
0	0	0	13	2	1	3760	57205	60	1011	1039	489
0	0	0	13	3	1	4155	57200	65	1052	1044	241
0	0	0	13	4	1	4535	57205	65	1134	1147	208
0	0	0	13	5	1	4915	57205	65	972	984	200
0	0	0	13	6	1	5285	57195	65	1043	1056	225
0	0	0	13	1	2	3400	57565	60	989	965	239
0	0	0	13	2	2	3790	57580	65	963	1034	504
0	0	0	13	3	2	4160	57600	75	945	958	186
0	0	0	13	4	2	4545	57600	80	892	868	252
0	0	0	13	5	2	4880	57595	75	953	991	331
0	0	0	13	6	2	5270	57570	60	1040	1062	203
0	0	0	13	1	3	3405	57945	60	1018	1044	239
0	0	0	13	2	3	3790	57960	70	929	939	188
0	0	0	13	3	3	4190	57965	90	790	772	237

0	0	0	13	4	3	4530	57980	95	807	815	159
0	0	0	13	5	3	4865	57955	80	730	725	186
0	0	0	13	6	3	5265	57955	65	1073	1061	239
0	0	0	13	1	4	3400	58330	60	935	921	225
0	0	0	13	2	4	3805	58315	75	832	856	201
0	0	0	13	3	4	4170	58295	85	779	801	148
0	0	0	13	4	4	4525	58290	95	783	799	158
0	0	0	13	5	4	4880	58315	85	927	935	200
0	0	0	13	6	4	5265	58325	70	1039	978	301
0	0	0	13	1	5	3400	58700	60	901	835	306
0	0	0	13	2	5	3785	58695	70	1011	1160	913
0	0	0	13	3	5	4175	58680	75	890	912	265
0	0	0	13	4	5	4530	58670	75	808	821	152
0	0	0	13	5	5	4890	58690	70	817	816	208
0	0	0	13	6	5	5270	58715	65	1014	1017	164
0	0	0	13	1	6	3385	59090	60	998	1009	269
0	0	0	13	2	6	3765	59080	65	906	936	382
0	0	0	13	3	6	4160	59075	65	987	960	199
0	0	0	13	4	6	4535	59075	65	1013	1000	351
0	0	0	13	5	6	4930	59075	55	1002	1031	175
0	0	0	13	6	6	5285	59095	65	943	939	213
0	0	0	14	1	1	3400	61085	115	159	168	76
0	0	0	14	2	1	3790	61100	115	185	193	76
0	0	0	14	3	1	4165	61105	105	161	172	74
0	0	0	14	4	1	4545	61110	100	154	170	72
0	0	0	14	5	1	4925	61110	105	186	196	76
0	0	0	14	6	1	5295	61090	110	177	192	93
0	0	0	14	1	2	3410	61465	105	164	173	69
0	0	0	14	2	2	3810	61495	115	168	171	64
0	0	0	14	3	2	4190	61500	110	150	162	67
0	0	0	14	4	2	4545	61500	110	129	136	50
0	0	0	14	5	2	4895	61480	105	149	162	71
-50	0	0	14	6	2	5265	61480	70	49	54	21
0	0	0	14	1	3	3425	61840	90	205	210	67
0	0	0	14	2	3	3820	61855	105	184	194	71
0	0	0	14	3	3	4200	61865	110	161	165	63
0	0	0	14	4	3	4525	61885	110	168	212	148
0	0	0	14	5	3	4895	61855	110	145	151	58
-50	0	0	14	6	3	5265	61855	70	54	58	27
0	0	0	14	1	4	3430	62225	105	336	351	119
0	0	0	14	2	4	3825	62220	110	246	259	117
0	0	0	14	3	4	4205	62205	120	185	200	100
0	0	0	14	4	4	4525	62190	110	178	217	161
0	0	0	14	5	4	4865	62215	80	149	174	133
0	0	0	14	6	4	5260	62265	40	129	176	133
0	0	0	14	1	5	3420	62605	110	235	281	164
0	0	0	14	2	5	3810	62600	120	255	294	166
0	0	0	14	3	5	4185	62575	120	212	268	196

0	0	0	14	4	5	4535	62565	110	220	254	147
0	0	0	14	5	5	4885	62570	80	216	258	156
0	0	0	14	6	5	5295	62620	90	174	191	111
0	0	0	14	1	6	3410	62980	110	268	284	137
0	0	0	14	2	6	3785	62965	110	256	280	147
0	0	0	14	3	6	4170	62960	110	244	271	154
0	0	0	14	4	6	4545	62960	105	245	290	165
0	0	0	14	5	6	4925	62965	100	245	330	593
0	0	0	14	6	6	5315	62965	70	199	265	213
0	0	0	15	1	1	7305	14270	90	478	492	222
0	0	0	15	2	1	7695	14275	85	423	460	195
0	0	0	15	3	1	8080	14280	85	588	598	238
0	0	0	15	4	1	8465	14285	85	532	543	209
0	0	0	15	5	1	8855	14285	95	450	477	230
0	0	0	15	6	1	9235	14275	90	596	659	425
0	0	0	15	1	2	7310	14655	90	613	627	299
0	0	0	15	2	2	7705	14665	100	405	439	315
0	0	0	15	3	2	8085	14680	100	497	525	286
0	0	0	15	4	2	8465	14680	95	508	525	210
0	0	0	15	5	2	8840	14675	105	512	548	675
0	0	0	15	6	2	9230	14660	85	699	819	525
0	0	0	15	1	3	7315	15040	85	710	1102	2400
0	0	0	15	2	3	7715	15045	100	498	525	264
0	0	0	15	3	3	8105	15055	115	401	420	233
0	0	0	15	4	3	8455	15070	125	389	427	232
0	0	0	15	5	3	8830	15045	100	578	605	307
0	0	0	15	6	3	9225	15040	85	638	734	451
0	0	0	15	1	4	7315	15430	95	806	845	413
0	0	0	15	2	4	7710	15430	95	615	655	333
0	0	0	15	3	4	8100	15420	110	478	504	319
0	0	0	15	4	4	8460	15405	110	428	456	239
0	0	0	15	5	4	8825	15425	110	428	465	276
0	0	0	15	6	4	9220	15435	90	701	755	436
0	0	0	15	1	5	7315	15820	95	850	926	476
0	0	0	15	2	5	7705	15810	105	665	700	408
0	0	0	15	3	5	8085	15800	100	1452	2279	2507
0	0	0	15	4	5	8460	15800	90	656	697	333
0	0	0	15	5	5	8840	15800	110	374	420	296
0	0	0	15	6	5	9225	15825	85	704	834	448
0	0	0	15	1	6	7310	16200	100	827	810	428
0	0	0	15	2	6	7690	16190	90	927	949	447
0	0	0	15	3	6	8080	16185	85	961	1037	414
0	0	0	15	4	6	8470	16190	90	836	924	543
0	0	0	15	5	6	8860	16185	90	770	851	535
0	0	0	15	6	6	9235	16190	100	656	706	489
0	0	0	16	1	1	7320	18180	85	3532	3578	782
0	0	0	16	2	1	7705	18195	95	3377	3271	1831
0	0	0	16	3	1	8085	18200	90	3166	3114	1433

0	0	0	16	4	1	8475	18200	85	3214	3399	1060
0	0	0	16	5	1	8855	18200	85	3154	3289	1048
0	0	0	16	6	1	9225	18185	85	3338	3564	1546
0	0	0	16	1	2	7330	18560	85	3437	3550	1182
0	0	0	16	2	2	7720	18580	95	2600	2675	886
0	0	0	16	3	2	8095	18590	95	2606	2709	946
0	0	0	16	4	2	8470	18595	100	2431	2538	1536
0	0	0	16	5	2	8830	18585	95	3032	3100	1272
0	0	0	16	6	2	9215	18565	90	3078	3165	1773
0	0	0	16	1	3	7335	18940	85	3598	3656	1161
0	0	0	16	2	3	7730	18950	95	2406	2584	891
0	0	0	16	3	3	8120	18960	110	1980	2096	1081
0	0	0	16	4	3	8455	18975	105	2089	2247	856
0	0	0	16	5	3	8815	18950	100	2067	2241	1053
0	0	0	16	6	3	9205	18945	90	2888	2927	1189
0	0	0	16	1	4	7340	19325	85	3416	3496	853
0	0	0	16	2	4	7730	19320	95	2574	2760	968
0	0	0	16	3	4	8115	19305	100	2201	2400	959
0	0	0	16	4	4	8455	19285	100	1936	2175	920
0	0	0	16	5	4	8820	19315	100	2164	2364	1055
0	0	0	16	6	4	9205	19325	85	3084	3143	975
0	0	0	16	1	5	7335	19710	85	3578	3762	968
0	0	0	16	2	5	7720	19700	90	2732	2743	860
0	0	0	16	3	5	8095	19685	95	2451	2768	1340
0	0	0	16	4	5	8465	19680	95	2198	2322	870
0	0	0	16	5	5	8835	19695	95	2453	2550	958
0	0	0	16	6	5	9210	19715	90	2997	3102	1270
0	0	0	16	1	6	7320	20090	90	3543	3357	1439
0	0	0	16	2	6	7705	20080	85	3645	3749	1036
0	0	0	16	3	6	8085	20075	85	3368	3517	1066
0	0	0	16	4	6	8470	20070	85	3301	3352	1032
0	0	0	16	5	6	8850	20075	85	3327	3418	1012
0	0	0	16	6	6	9230	20090	90	3372	3339	1438
0	0	0	17	1	1	7315	22075	105	1034	1071	538
0	0	0	17	2	1	7695	22090	105	1013	1046	442
0	0	0	17	3	1	8080	22095	100	669	736	407
0	0	0	17	4	1	8470	22100	105	722	776	361
0	0	0	17	5	1	8850	22095	105	753	811	416
0	0	0	17	6	1	9225	22080	105	806	897	593
0	0	0	17	1	2	7325	22440	110	656	674	443
0	0	0	17	2	2	7715	22465	105	664	724	379
0	0	0	17	3	2	8090	22485	115	464	560	401
0	0	0	17	4	2	8465	22490	115	471	531	302
0	0	0	17	5	2	8830	22480	110	525	581	373
0	0	0	17	6	2	9210	22460	105	521	604	408
0	0	0	17	1	3	7330	22840	105	793	810	346
0	0	0	17	2	3	7725	22845	110	600	691	725
0	0	0	17	3	3	8105	22860	120	498	545	297

0	0	0	17	4	3	8455	22875	120	401	493	318
0	0	0	17	5	3	8815	22850	115	477	544	321
0	0	0	17	6	3	9205	22845	110	431	519	359
0	0	0	17	1	4	7330	23225	105	887	881	371
0	0	0	17	2	4	7720	23220	105	894	958	542
0	0	0	17	3	4	8110	23205	120	512	600	445
0	0	0	17	4	4	8455	23190	125	388	437	231
0	0	0	17	5	4	8815	23215	115	459	515	276
0	0	0	17	6	4	9205	23225	105	661	732	392
0	0	0	17	1	5	7330	23615	105	1068	1132	1029
0	0	0	17	2	5	7715	23600	110	736	812	472
0	0	0	17	3	5	8090	23585	105	614	653	324
0	0	0	17	4	5	8465	23585	110	563	625	339
0	0	0	17	5	5	8835	23595	115	567	625	383
0	0	0	17	6	5	9215	23615	105	962	1060	550
0	0	0	17	1	6	7320	23985	105	1368	1403	606
0	0	0	17	2	6	7700	23980	105	1178	1218	552
0	0	0	17	3	6	8080	23975	100	1102	1135	489
0	0	0	17	4	6	8465	23975	105	973	1007	452
0	0	0	17	5	6	8850	23975	105	780	840	374
0	0	0	17	6	6	9225	23990	110	925	1059	811
0	0	0	18	1	1	7310	25975	90	4653	4664	2001
0	0	0	18	2	1	7695	25985	90	4540	4485	655
0	0	0	18	3	1	8080	25990	95	4411	4345	709
0	0	0	18	4	1	8465	25995	90	4260	4276	554
0	0	0	18	5	1	8845	25990	90	4198	4207	495
0	0	0	18	6	1	9220	25975	95	4098	3998	765
0	0	0	18	1	2	7320	26355	90	4729	4603	852
0	0	0	18	2	2	7710	26365	95	4541	4552	576
0	0	0	18	3	2	8090	26380	100	4232	4234	926
0	0	0	18	4	2	8465	26390	105	4205	4210	661
0	0	0	18	5	2	8830	26375	100	4141	4208	2093
0	0	0	18	6	2	9210	26360	95	4102	4158	535
0	0	0	18	1	3	7325	26740	95	4769	4707	842
0	0	0	18	2	3	7720	26745	100	4519	4443	894
0	0	0	18	3	3	8110	26755	110	4245	4082	1101
0	0	0	18	4	3	8450	26775	110	4175	4157	680
0	0	0	18	5	3	8815	26750	110	4142	3889	1454
0	0	0	18	6	3	9200	26745	95	4095	4282	2279
0	0	0	18	1	4	7325	27125	90	4798	4747	756
0	0	0	18	2	4	7720	27120	100	4548	4447	905
0	0	0	18	3	4	8105	27105	110	4195	3981	1245
0	0	0	18	4	4	8455	27095	115	4255	4318	1894
0	0	0	18	5	4	8815	27115	105	4235	4238	588
0	0	0	18	6	4	9200	27125	95	4086	4176	1693
0	0	0	18	1	5	7320	27510	95	4696	4599	893
0	0	0	18	2	5	7710	27500	100	4584	4260	1199
0	0	0	18	3	5	8085	27485	100	4304	4204	833

0	0	0	18	4	5	8465	27485	100	4356	4348	666
0	0	0	18	5	5	8830	27495	100	4127	4071	878
0	0	0	18	6	5	9210	27515	95	4212	4244	568
0	0	0	18	1	6	7310	27895	95	4652	4703	790
0	0	0	18	2	6	7695	27880	90	4812	4882	1684
0	0	0	18	3	6	8080	27875	95	4615	4608	673
0	0	0	18	4	6	8465	27870	95	4487	4369	934
0	0	0	18	5	6	8850	27875	90	4335	4422	945
0	0	0	18	6	6	9225	27890	90	4315	4298	636
0	0	0	19	1	1	7285	29865	110	5709	5049	2428
0	0	0	19	2	1	7680	29870	110	5463	4960	2054
0	0	0	19	3	1	8070	29870	95	5554	5647	851
0	0	0	19	4	1	8465	29870	95	5193	5307	668
0	0	0	19	5	1	8850	29865	95	5296	5311	582
0	0	0	19	6	1	9245	29860	115	5308	4755	2232
0	0	0	19	1	2	7300	30250	110	5602	5080	1915
0	0	0	19	2	2	7695	30265	130	5355	4674	2023
0	0	0	19	3	2	8080	30265	115	5305	5296	1070
0	0	0	19	4	2	8465	30275	120	5046	4826	1461
0	0	0	19	5	2	8840	30270	130	5057	4381	2027
0	0	0	19	6	2	9235	30255	110	5205	4874	2666
0	0	0	19	1	3	7300	30640	105	5651	5504	1148
0	0	0	19	2	3	7695	30645	110	5449	5397	928
0	0	0	19	3	3	8090	30655	135	5250	5388	1716
0	0	0	19	4	3	8455	30665	140	4967	4532	1740
0	0	0	19	5	3	8830	30650	135	4970	4733	1447
0	0	0	19	6	3	9235	30645	100	5195	5132	861
0	0	0	19	1	4	7300	31030	95	5588	5595	536
0	0	0	19	2	4	7695	31030	110	5393	5339	1151
0	0	0	19	3	4	8095	31020	140	5064	4390	1906
0	0	0	19	4	4	8460	31010	140	4882	4557	1735
0	0	0	19	5	4	8830	31025	130	4880	4465	1721
0	0	0	19	6	4	9235	31035	105	5157	5159	728
0	0	0	19	1	5	7295	31420	95	5474	5520	745
0	0	0	19	2	5	7695	31415	125	5204	4652	1864
0	0	0	19	3	5	8080	31405	130	5222	4708	2212
0	0	0	19	4	5	8460	31410	120	5195	4939	1537
0	0	0	19	5	5	8845	31415	130	4803	4052	2094
0	0	0	19	6	5	9240	31430	100	5104	5069	857
0	0	0	19	1	6	7280	31820	105	5566	5377	1511
0	0	0	19	2	6	7675	31805	105	5458	5318	1343
0	0	0	19	3	6	8075	31800	95	5403	5432	590
0	0	0	19	4	6	8470	31795	110	5216	4744	1681
0	0	0	19	5	6	8860	31800	110	4963	4518	1781
0	0	0	19	6	6	9245	31815	100	5288	5121	1207
0	0	0	20	1	1	7310	33780	90	5682	5505	2014
0	0	0	20	2	1	7695	33795	90	5639	5553	1268
0	0	0	20	3	1	8080	33800	90	5641	5598	1065

0	0	0	20	4	1	8465	33805	90	5666	5970	3488
0	0	0	20	5	1	8845	33800	90	5457	5506	926
0	0	0	20	6	1	9220	33785	90	5566	5617	1058
0	0	0	20	1	2	7325	34160	90	5513	5504	902
0	0	0	20	2	2	7710	34175	95	5473	6326	4476
0	0	0	20	3	2	8090	34190	100	5244	5102	1026
0	0	0	20	4	2	8460	34195	100	4948	5049	832
0	0	0	20	5	2	8825	34185	100	4833	4824	1485
0	0	0	20	6	2	9205	34165	90	5329	5400	857
0	0	0	20	1	3	7330	34540	90	5526	5449	1011
0	0	0	20	2	3	7725	34545	100	5082	4925	1440
0	0	0	20	3	3	8110	34560	110	4838	4560	1418
0	0	0	20	4	3	8445	34580	110	4604	4586	1000
0	0	0	20	5	3	8810	34550	105	4799	4874	709
0	0	0	20	6	3	9200	34545	90	5037	4984	910
0	0	0	20	1	4	7330	34925	90	5654	5452	1117
0	0	0	20	2	4	7720	34915	95	5128	5126	989
0	0	0	20	3	4	8105	34905	105	5099	5087	708
0	0	0	20	4	4	8455	34890	110	4784	4708	1123
0	0	0	20	5	4	8810	34915	100	4615	4766	883
0	0	0	20	6	4	9200	34925	90	5161	5238	993
0	0	0	20	1	5	7325	35310	90	5594	5685	2529
0	0	0	20	2	5	7710	35300	95	5552	5680	1724
0	0	0	20	3	5	8085	35285	95	5292	5270	727
0	0	0	20	4	5	8460	35280	95	5344	5315	730
0	0	0	20	5	5	8830	35295	100	5224	5104	1627
0	0	0	20	6	5	9205	35315	90	5579	5660	808
0	0	0	20	1	6	7310	35690	90	5976	6049	2199
0	0	0	20	2	6	7695	35680	90	5656	5514	1122
0	0	0	20	3	6	8080	35675	90	5694	5688	940
0	0	0	20	4	6	8460	35670	90	5520	5401	1146
0	0	0	20	5	6	8845	35675	90	5874	5819	1186
0	0	0	20	6	6	9220	35690	90	5967	5836	1190
0	0	0	21	1	1	7305	37665	90	73	81	37
0	0	0	21	2	1	7715	37665	60	73	79	33
0	0	0	21	3	1	8075	37685	105	69	81	86
0	0	0	21	4	1	8465	37690	100	79	86	58
0	0	0	21	5	1	8860	37665	70	77	79	34
0	0	0	21	6	1	9220	37680	110	81	91	58
0	0	0	21	1	2	7345	38020	35	61	71	35
0	0	0	21	2	2	7725	38050	70	66	72	30
0	0	0	21	3	2	8080	38060	85	77	85	36
0	0	0	21	4	2	8440	38065	85	72	82	49
0	0	0	21	5	2	8845	38065	105	80	99	203
0	0	0	21	6	2	9210	38055	110	88	94	41
0	0	0	21	1	3	7330	38415	60	71	75	30
0	0	0	21	2	3	7730	38425	80	70	73	28
0	0	0	21	3	3	8130	38430	60	70	74	30

0	0	0	21	4	3	8475	38450	70	74	77	33
0	0	0	21	5	3	8840	38430	65	80	81	32
0	0	0	21	6	3	9215	38410	70	75	83	44
0	0	0	21	1	4	7325	38825	105	76	82	35
0	0	0	21	2	4	7735	38815	80	72	78	34
0	0	0	21	3	4	8120	38775	75	65	72	34
0	0	0	21	4	4	8445	38760	55	69	73	33
0	0	0	21	5	4	8835	38800	85	70	80	39
0	0	0	21	6	4	9220	38825	85	78	86	44
0	0	0	21	1	5	7300	39215	80	81	92	61
0	0	0	21	2	5	7720	39170	50	85	92	41
-50	0	0	21	3	5	8090	39160	70	48	53	21
0	0	0	21	4	5	8460	39150	70	66	135	567
0	0	0	21	5	5	8840	39180	80	69	73	32
0	0	0	21	6	5	9220	39225	85	85	93	50
0	0	0	21	1	6	7305	39590	115	84	89	37
0	0	0	21	2	6	7670	39575	75	59	66	29
0	0	0	21	3	6	8065	39570	75	71	72	27
0	0	0	21	4	6	8440	39550	60	59	64	26
0	0	0	21	5	6	8850	39580	125	71	92	286
0	0	0	21	6	6	9225	39560	35	61	62	24
0	0	0	22	1	1	7310	41575	100	4938	4611	1313
0	0	0	22	2	1	7695	41590	95	4645	4787	1070
0	0	0	22	3	1	8075	41595	95	4760	4808	555
0	0	0	22	4	1	8465	41595	100	4638	4611	1179
0	0	0	22	5	1	8850	41595	100	4829	4749	1030
0	0	0	22	6	1	9220	41580	100	5157	5104	1825
0	0	0	22	1	2	7320	41955	100	4755	4549	1164
0	0	0	22	2	2	7710	41970	105	4647	4690	640
0	0	0	22	3	2	8095	41985	105	4513	4602	606
0	0	0	22	4	2	8460	41990	110	4373	4303	1120
0	0	0	22	5	2	8830	41980	105	4711	4809	688
0	0	0	22	6	2	9205	41960	100	5086	4968	1208
0	0	0	22	1	3	7325	42340	100	4386	4182	1032
0	0	0	22	2	3	7725	42340	105	4256	4333	682
0	0	0	22	3	3	8110	42355	115	4469	4543	799
0	0	0	22	4	3	8450	42375	120	4475	4390	1219
0	0	0	22	5	3	8805	42350	110	4365	4382	1027
0	0	0	22	6	3	9200	42340	95	4952	5027	637
0	0	0	22	1	4	7325	42725	95	4506	4483	450
0	0	0	22	2	4	7720	42725	105	4432	4446	739
0	0	0	22	3	4	8100	42710	115	4502	4513	619
0	0	0	22	4	4	8455	42685	115	4438	4471	691
0	0	0	22	5	4	8810	42720	115	4433	4286	1095
0	0	0	22	6	4	9200	42735	100	4869	4919	2409
0	0	0	22	1	5	7320	43110	95	4460	4487	543
0	0	0	22	2	5	7710	43105	105	4303	4678	3288
0	0	0	22	3	5	8085	43090	105	4524	4580	674

0	0	0	22	4	5	8460	43080	105	4417	4451	560
0	0	0	22	5	5	8835	43095	105	4473	4705	1744
0	0	0	22	6	5	9205	43115	100	4559	4609	1202
0	0	0	22	1	6	7310	43490	95	4499	4508	545
0	0	0	22	2	6	7690	43480	95	4292	4372	596
0	0	0	22	3	6	8080	43475	95	4453	4533	462
0	0	0	22	4	6	8465	43475	100	4394	4202	1119
0	0	0	22	5	6	8850	43475	95	4502	4558	509
0	0	0	22	6	6	9225	43490	100	4942	4722	1293
0	0	0	23	1	1	7310	45485	90	6015	5628	1642
0	0	0	23	2	1	7690	45490	85	5544	5534	936
0	0	0	23	3	1	8080	45500	90	6380	6303	1274
0	0	0	23	4	1	8460	45500	95	8533	8536	1906
0	0	0	23	5	1	8845	45500	95	8461	8428	3079
0	0	0	23	6	1	9215	45485	90	8418	8277	1961
0	0	0	23	1	2	7325	45860	90	5582	5596	2068
0	0	0	23	2	2	7710	45875	95	5482	5442	772
0	0	0	23	3	2	8085	45890	100	5851	5775	1536
0	0	0	23	4	2	8455	45895	100	7035	7045	1588
0	0	0	23	5	2	8825	45885	100	6876	6651	1927
0	0	0	23	6	2	9205	45865	90	7880	7871	1872
0	0	0	23	1	3	7330	46240	90	5500	5310	1396
0	0	0	23	2	3	7720	46245	95	5153	5148	807
0	0	0	23	3	3	8110	46260	105	5332	5310	899
0	0	0	23	4	3	8450	46285	110	5821	5750	1353
0	0	0	23	5	3	8805	46250	100	6484	6552	1581
0	0	0	23	6	3	9195	46245	95	7413	7338	1576
0	0	0	23	1	4	7330	46630	90	5537	5308	1235
0	0	0	23	2	4	7720	46620	95	5269	5289	1005
0	0	0	23	3	4	8100	46610	105	5374	5298	991
0	0	0	23	4	4	8455	46595	110	5663	5520	1511
0	0	0	23	5	4	8805	46620	105	6158	6131	1254
0	0	0	23	6	4	9195	46630	95	7326	7190	1571
0	0	0	23	1	5	7325	47015	90	5584	5484	1603
0	0	0	23	2	5	7705	47005	100	5069	4668	1571
0	0	0	23	3	5	8090	46990	100	5473	5292	1304
0	0	0	23	4	5	8460	46985	100	6187	5997	1365
0	0	0	23	5	5	8825	47000	105	6418	6254	1909
0	0	0	23	6	5	9205	47020	90	7846	7741	1464
0	0	0	23	1	6	7310	47395	90	5688	5482	1072
0	0	0	23	2	6	7695	47375	90	5368	5163	1169
0	0	0	23	3	6	8080	47370	90	6068	6020	1165
0	0	0	23	4	6	8460	47370	90	6901	6764	1330
0	0	0	23	5	6	8840	47375	90	7281	7230	1445
0	0	0	23	6	6	9220	47390	90	10768	11046	3034
0	0	0	24	1	1	7305	49375	95	4930	5147	1531
0	0	0	24	2	1	7685	49390	95	4507	4724	1270
0	0	0	24	3	1	8070	49395	95	4456	4775	1269

0	0	0	24	4	1	8460	49400	95	4793	5056	1437
0	0	0	24	5	1	8845	49395	100	4699	4963	1872
0	0	0	24	6	1	9215	49385	100	5202	5455	2172
0	0	0	24	1	2	7315	49760	100	4226	4060	1556
0	0	0	24	2	2	7710	49770	105	3880	4013	1315
0	0	0	24	3	2	8090	49790	105	3706	3900	1418
0	0	0	24	4	2	8450	49790	105	3844	4105	1340
0	0	0	24	5	2	8825	49780	110	4012	4056	1964
0	0	0	24	6	2	9205	49760	100	4889	5099	1992
0	0	0	24	1	3	7320	50140	95	4627	4669	860
0	0	0	24	2	3	7710	50145	105	3756	3836	1044
0	0	0	24	3	3	8100	50155	115	3405	3577	957
0	0	0	24	4	3	8445	50175	120	3276	3548	2483
0	0	0	24	5	3	8805	50150	110	3795	4006	1440
0	0	0	24	6	3	9195	50145	100	4854	5104	1848
0	0	0	24	1	4	7320	50530	100	4375	4151	1513
0	0	0	24	2	4	7715	50525	110	3555	3461	1479
0	0	0	24	3	4	8100	50505	115	3367	3394	894
0	0	0	24	4	4	8440	50495	115	3611	4026	3538
0	0	0	24	5	4	8810	50520	110	3770	3933	1327
0	0	0	24	6	4	9200	50530	100	4891	5168	2377
0	0	0	24	1	5	7315	50915	95	4363	4449	1058
0	0	0	24	2	5	7705	50905	105	3930	3836	979
0	0	0	24	3	5	8085	50890	105	3808	3709	941
0	0	0	24	4	5	8450	50880	105	3776	3849	1162
0	0	0	24	5	5	8830	50895	105	4282	4345	1323
0	0	0	24	6	5	9205	50920	100	5089	5638	4010
0	0	0	24	1	6	7300	51285	95	4642	4646	1142
0	0	0	24	2	6	7685	51280	95	4256	4417	2646
0	0	0	24	3	6	8070	51275	90	4471	4584	1355
0	0	0	24	4	6	8460	51275	100	4419	4351	1404
0	0	0	24	5	6	8845	51280	95	4749	4925	1167
0	0	0	24	6	6	9220	51290	95	5087	5336	1505
0	0	0	25	1	1	7295	53275	95	192	355	2222
0	0	0	25	2	1	7685	53285	95	179	193	87
0	0	0	25	3	1	8070	53295	105	154	175	89
0	0	0	25	4	1	8460	53295	100	171	191	97
0	0	0	25	5	1	8840	53290	100	173	199	109
0	0	0	25	6	1	9215	53280	100	174	201	108
0	0	0	25	1	2	7310	53655	95	175	335	2122
0	0	0	25	2	2	7705	53670	105	160	174	82
0	0	0	25	3	2	8080	53685	105	125	145	68
0	0	0	25	4	2	8450	53690	105	144	159	69
0	0	0	25	5	2	8820	53680	110	138	155	90
0	0	0	25	6	2	9200	53660	105	166	193	119
0	0	0	25	1	3	7315	54040	100	156	172	82
0	0	0	25	2	3	7710	54050	105	144	151	65
0	0	0	25	3	3	8105	54060	120	134	144	76

0	0	0	25	4	3	8435	54075	120	122	136	64
0	0	0	25	5	3	8805	54055	115	125	144	77
0	0	0	25	6	3	9195	54040	100	187	205	109
0	0	0	25	1	4	7315	54430	95	179	213	341
0	0	0	25	2	4	7715	54420	110	151	160	74
0	0	0	25	3	4	8095	54410	120	133	149	70
0	0	0	25	4	4	8445	54395	120	128	137	63
0	0	0	25	5	4	8805	54420	115	124	145	128
0	0	0	25	6	4	9200	54430	105	164	176	88
0	0	0	25	1	5	7310	54820	95	178	191	85
0	0	0	25	2	5	7705	54810	105	132	143	118
0	0	0	25	3	5	8080	54790	110	147	167	128
0	0	0	25	4	5	8450	54785	115	140	198	994
0	0	0	25	5	5	8825	54800	110	137	150	81
0	0	0	25	6	5	9210	54820	95	162	176	87
0	0	0	25	1	6	7300	55190	100	174	192	92
0	0	0	25	2	6	7690	55185	95	145	165	86
0	0	0	25	3	6	8070	55185	100	160	168	74
0	0	0	25	4	6	8460	55180	95	175	186	87
0	0	0	25	5	6	8845	55185	95	154	178	92
0	0	0	25	6	6	9220	55195	95	142	156	76
0	0	0	26	1	1	7315	57180	85	5619	5636	884
0	0	0	26	2	1	7695	57195	85	5249	5397	1014
0	0	0	26	3	1	8080	57200	85	5238	5369	1007
0	0	0	26	4	1	8465	57205	90	5143	4988	1329
0	0	0	26	5	1	8845	57200	90	5302	5266	1057
0	0	0	26	6	1	9220	57185	90	5562	5393	1373
0	0	0	26	1	2	7330	57560	85	5346	5587	2699
0	0	0	26	2	2	7710	57575	95	4881	4831	909
0	0	0	26	3	2	8090	57590	100	4684	4785	2046
0	0	0	26	4	2	8465	57600	100	4475	4482	1174
0	0	0	26	5	2	8825	57585	100	4724	4669	1129
0	0	0	26	6	2	9205	57565	90	5067	5093	1249
0	0	0	26	1	3	7335	57940	90	4836	4707	1352
0	0	0	26	2	3	7730	57950	100	4456	4328	1203
0	0	0	26	3	3	8115	57955	110	4124	4062	1173
0	0	0	26	4	3	8450	57975	115	4058	4053	978
0	0	0	26	5	3	8805	57950	105	5689	10961	14652
0	0	0	26	6	3	9200	57940	95	4766	4718	1180
0	0	0	26	1	4	7335	58325	90	4949	4883	1389
0	0	0	26	2	4	7725	58315	100	4349	4216	1230
0	0	0	26	3	4	8110	58305	110	3865	3731	1317
0	0	0	26	4	4	8450	58290	110	3938	3926	1122
0	0	0	26	5	4	8810	58315	105	4231	4268	2052
0	0	0	26	6	4	9200	58325	90	4835	4899	1002
0	0	0	26	1	5	7330	58705	90	4598	4482	1256
0	0	0	26	2	5	7710	58695	95	4339	4372	881
0	0	0	26	3	5	8090	58685	100	4047	4056	1070

0	0	0	26	4	5	8470	58680	100	4069	3992	1033
0	0	0	26	5	5	8830	58695	100	4416	4312	1430
0	0	0	26	6	5	9205	58715	95	4774	4762	1123
0	0	0	26	1	6	7310	59085	85	4793	4803	894
0	0	0	26	2	6	7695	59080	85	4157	4317	1382
0	0	0	26	3	6	8080	59075	85	4370	4429	750
0	0	0	26	4	6	8465	59070	85	4651	4702	835
0	0	0	26	5	6	8845	59080	90	4825	4797	1107
0	0	0	26	6	6	9220	59095	90	4734	4601	1194
0	0	0	27	1	1	7300	61075	90	5465	5486	648
0	0	0	27	2	1	7685	61090	95	5425	5486	773
0	0	0	27	3	1	8065	61095	95	5058	5003	904
0	0	0	27	4	1	8455	61100	95	4607	4637	639
0	0	0	27	5	1	8830	61095	95	4753	4774	722
0	0	0	27	6	1	9205	61080	90	4781	4788	658
0	0	0	27	1	2	7310	61460	95	5499	5523	633
0	0	0	27	2	2	7700	61480	100	4603	4444	1193
0	0	0	27	3	2	8080	61490	100	4282	4347	779
0	0	0	27	4	2	8450	61495	105	4233	4263	780
0	0	0	27	5	2	8815	61480	100	4347	4310	735
0	0	0	27	6	2	9195	61465	100	4652	4365	1305
0	0	0	27	1	3	7315	61840	95	5122	5106	551
0	0	0	27	2	3	7715	61850	100	4167	4080	708
0	0	0	27	3	3	8095	61865	110	4106	4024	837
0	0	0	27	4	3	8440	61885	115	4019	3944	759
0	0	0	27	5	3	8795	61850	105	4131	4101	793
0	0	0	27	6	3	9190	61845	100	4585	4282	1260
0	0	0	27	1	4	7315	62225	90	4752	4778	586
0	0	0	27	2	4	7715	62220	100	4215	4148	748
0	0	0	27	3	4	8095	62205	115	4139	4017	1046
0	0	0	27	4	4	8435	62200	110	4091	4035	762
0	0	0	27	5	4	8805	62220	110	4473	4349	971
0	0	0	27	6	4	9185	62225	95	4623	4495	770
0	0	0	27	1	5	7310	62610	95	4785	4790	650
0	0	0	27	2	5	7695	62605	105	4810	4734	790
0	0	0	27	3	5	8075	62585	105	4428	4387	979
0	0	0	27	4	5	8450	62580	105	4441	4383	921
0	0	0	27	5	5	8820	62595	105	4693	4709	1833
0	0	0	27	6	5	9195	62615	95	4943	4908	655
0	0	0	27	1	6	7295	62995	95	5430	5264	835
0	0	0	27	2	6	7680	62975	95	5115	5084	664
0	0	0	27	3	6	8065	62970	90	5273	5181	623
0	0	0	27	4	6	8450	62965	95	5257	5259	718
0	0	0	27	5	6	8835	62970	95	5321	5281	660
0	0	0	27	6	6	9210	62985	90	5398	5304	732
0	0	0	28	1	1	11200	14270	105	3804	4083	2843
0	0	0	28	2	1	11585	14285	110	3287	3101	985
0	0	0	28	3	1	11970	14290	110	2936	2965	1520

0	0	0	28	4	1	12360	14290	110	2913	2788	804
0	0	0	28	5	1	12745	14290	110	3107	3010	943
0	0	0	28	6	1	13120	14275	110	3237	3184	1739
0	0	0	28	1	2	11210	14650	110	3881	3644	1229
0	0	0	28	2	2	11600	14665	115	3520	3536	625
0	0	0	28	3	2	11980	14680	115	3217	3282	648
0	0	0	28	4	2	12360	14685	115	3196	3278	1277
0	0	0	28	5	2	12730	14675	120	2797	2670	861
0	0	0	28	6	2	13110	14655	115	3110	3041	785
0	0	0	28	1	3	11215	15040	110	4167	3905	1363
0	0	0	28	2	3	11610	15040	115	3705	3683	601
0	0	0	28	3	3	12000	15055	120	3490	3371	847
0	0	0	28	4	3	12350	15070	130	2968	2737	954
0	0	0	28	5	3	12710	15045	120	3014	2902	723
0	0	0	28	6	3	13105	15040	110	3415	3298	861
0	0	0	28	1	4	11215	15425	110	4139	3859	1236
0	0	0	28	2	4	11610	15420	115	4583	4688	993
0	0	0	28	3	4	11995	15410	125	3731	3689	829
0	0	0	28	4	4	12350	15400	125	3296	3281	703
0	0	0	28	5	4	12710	15420	120	3501	3419	1142
0	0	0	28	6	4	13105	15430	110	3828	3707	1016
0	0	0	28	1	5	11210	15810	110	3935	3750	1196
0	0	0	28	2	5	11600	15800	115	4209	4151	818
0	0	0	28	3	5	11980	15790	115	4189	4203	661
0	0	0	28	4	5	12355	15785	115	4213	4322	2116
0	0	0	28	5	5	12730	15795	115	4206	4252	1038
0	0	0	28	6	5	13110	15815	110	4301	4224	1449
0	0	0	28	1	6	11200	16195	110	4776	4686	1752
0	0	0	28	2	6	11580	16170	105	5464	5447	1185
0	0	0	28	3	6	11975	16170	105	5415	5483	1061
0	0	0	28	4	6	12360	16165	105	5764	5776	1007
0	0	0	28	5	6	12745	16175	105	5291	5357	2009
0	0	0	28	6	6	13125	16185	110	4536	4320	1348
0	0	0	29	1	1	11200	18170	95	2141	2436	1472
0	0	0	29	2	1	11585	18180	85	2359	2557	1124
0	0	0	29	3	1	11980	18190	85	2048	2293	1136
0	0	0	29	4	1	12360	18190	90	2557	2796	1501
0	0	0	29	5	1	12745	18185	90	1506	1781	1082
0	0	0	29	6	1	13120	18175	90	1686	1930	1081
0	0	0	29	1	2	11215	18550	90	2208	2588	1886
0	0	0	29	2	2	11600	18565	95	1777	2064	1116
0	0	0	29	3	2	11980	18580	100	2288	2767	1685
0	0	0	29	4	2	12360	18585	100	1425	1856	1366
0	0	0	29	5	2	12725	18570	110	1300	1604	1228
0	0	0	29	6	2	13110	18555	95	1320	1584	936
0	0	0	29	1	3	11220	18935	90	2343	2635	1577
0	0	0	29	2	3	11610	18940	95	1807	2171	1434
0	0	0	29	3	3	12000	18955	110	1021	1377	1071

0	0	0	29	4	3	12345	18970	110	934	1243	884
0	0	0	29	5	3	12710	18945	105	1047	1349	948
0	0	0	29	6	3	13105	18940	95	1542	1823	1055
0	0	0	29	1	4	11215	19320	85	2926	3131	1621
0	0	0	29	2	4	11610	19320	100	1585	2081	1427
0	0	0	29	3	4	12000	19305	110	878	1277	1057
0	0	0	29	4	4	12350	19295	115	767	1079	1239
0	0	0	29	5	4	12710	19320	105	1128	1522	1102
0	0	0	29	6	4	13105	19325	95	1536	1816	1146
0	0	0	29	1	5	11215	19710	90	2491	3052	1907
0	0	0	29	2	5	11600	19700	105	1989	2513	1687
0	0	0	29	3	5	11980	19685	100	1550	2058	1524
0	0	0	29	4	5	12355	19685	100	1155	1493	1066
0	0	0	29	5	5	12730	19695	100	1328	1707	1165
0	0	0	29	6	5	13110	19710	95	1654	2075	2276
0	0	0	29	1	6	11200	20085	90	3194	3485	2022
0	0	0	29	2	6	11590	20080	90	2757	3248	1955
0	0	0	29	3	6	11970	20075	90	2616	3160	1902
0	0	0	29	4	6	12360	20075	90	2307	2847	1831
0	0	0	29	5	6	12745	20075	85	2407	2783	1688
0	0	0	29	6	6	13125	20090	90	2076	2357	1403
0	0	0	30	1	1	11195	22070	105	5756	5723	1272
0	0	0	30	2	1	11580	22085	105	4101	4163	993
0	0	0	30	3	1	11965	22085	105	4039	4118	918
0	0	0	30	4	1	12355	22085	105	4324	4449	1975
0	0	0	30	5	1	12735	22085	105	4199	4284	905
0	0	0	30	6	1	13110	22070	105	4350	4270	995
0	0	0	30	1	2	11210	22445	105	5381	5257	1352
0	0	0	30	2	2	11600	22465	115	3503	3487	1161
0	0	0	30	3	2	11975	22480	115	3574	3699	1032
0	0	0	30	4	2	12355	22485	115	3746	3734	805
0	0	0	30	5	2	12725	22475	115	3683	3749	1056
0	0	0	30	6	2	13100	22455	110	3822	3699	1309
0	0	0	30	1	3	11210	22835	110	5392	5166	2113
0	0	0	30	2	3	11610	22840	115	4751	4761	1209
0	0	0	30	3	3	11995	22850	120	3851	3783	1069
0	0	0	30	4	3	12340	22865	120	3725	3682	1068
0	0	0	30	5	3	12705	22845	120	3458	3376	1101
0	0	0	30	6	3	13095	22835	105	4246	4258	815
0	0	0	30	1	4	11215	23220	105	6202	6287	1571
0	0	0	30	2	4	11605	23215	110	5251	5281	1498
0	0	0	30	3	4	11995	23205	120	4498	4436	1330
0	0	0	30	4	4	12345	23190	120	4035	4004	973
0	0	0	30	5	4	12710	23215	120	4150	4106	1526
0	0	0	30	6	4	13095	23220	105	4510	4661	2443
0	0	0	30	1	5	11210	23610	110	7018	6601	2752
0	0	0	30	2	5	11595	23595	115	5421	5357	1779
0	0	0	30	3	5	11975	23585	110	5510	5468	1369

0	0	0	30	4	5	12350	23580	115	5156	5106	1279
0	0	0	30	5	5	12725	23595	115	5351	5365	1349
0	0	0	30	6	5	13100	23610	105	6013	5967	1230
0	0	0	30	1	6	11195	23980	110	6508	6179	2344
0	0	0	30	2	6	11580	23975	105	6819	6928	1742
0	0	0	30	3	6	11970	23975	105	7016	7137	1773
0	0	0	30	4	6	12355	23970	105	7861	7944	2036
0	0	0	30	5	6	12740	23975	105	7707	8061	1878
0	0	0	30	6	6	13120	23985	105	7621	7805	1860
0	0	0	31	1	1	11200	25970	110	4044	3814	1492
0	0	0	31	2	1	11580	25980	110	3939	3968	2116
0	0	0	31	3	1	11965	25985	110	3554	3438	1286
0	0	0	31	4	1	12355	25985	105	3586	3670	1281
0	0	0	31	5	1	12740	25985	110	3153	3106	1129
0	0	0	31	6	1	13115	25970	110	2810	2698	964
0	0	0	31	1	2	11210	26345	110	3023	2998	1038
0	0	0	31	2	2	11595	26360	115	3098	3061	771
0	0	0	31	3	2	11975	26375	115	3152	3175	943
0	0	0	31	4	2	12350	26380	115	2911	2937	780
0	0	0	31	5	2	12725	26370	120	2969	2787	1143
0	0	0	31	6	2	13100	26350	110	2908	2830	911
0	0	0	31	1	3	11210	26735	110	3128	3022	1166
0	0	0	31	2	3	11605	26740	115	2846	2829	883
0	0	0	31	3	3	11995	26750	125	2610	2586	794
0	0	0	31	4	3	12345	26765	125	2526	2545	765
0	0	0	31	5	3	12710	26740	120	3029	2814	974
0	0	0	31	6	3	13095	26730	105	3151	3163	812
0	0	0	31	1	4	11210	27120	110	3369	3315	1183
0	0	0	31	2	4	11605	27120	115	2850	2907	913
0	0	0	31	3	4	12000	27105	125	2328	2304	779
0	0	0	31	4	4	12340	27090	125	2213	2279	816
0	0	0	31	5	4	12710	27115	120	2917	2797	983
0	0	0	31	6	4	13100	27120	110	3204	3070	1084
0	0	0	31	1	5	11205	27510	110	3291	3157	1235
0	0	0	31	2	5	11595	27500	115	2858	2834	799
0	0	0	31	3	5	11980	27485	115	2710	2742	859
0	0	0	31	4	5	12355	27485	115	2796	2838	797
0	0	0	31	5	5	12725	27490	115	2839	2832	839
0	0	0	31	6	5	13100	27510	110	3063	3011	1013
0	0	0	31	1	6	11195	27890	110	3437	3405	1195
0	0	0	31	2	6	11580	27875	105	3320	3467	984
0	0	0	31	3	6	11965	27875	105	3154	3210	822
0	0	0	31	4	6	12355	27870	105	3252	3333	893
0	0	0	31	5	6	12745	27875	110	3195	3213	1131
0	0	0	31	6	6	13120	27885	110	3253	3264	1464
0	0	0	32	1	1	11200	29875	80	380	394	202
0	0	0	32	2	1	11585	29885	75	429	450	179
0	0	0	32	3	1	11970	29890	75	408	439	179

0	0	0	32	4	1	12360	29885	70	354	375	158
0	0	0	32	5	1	12745	29885	75	406	427	249
0	0	0	32	6	1	13120	29875	80	346	363	182
0	0	0	32	1	2	11210	30255	75	449	452	148
0	0	0	32	2	2	11600	30265	80	336	346	227
0	0	0	32	3	2	11975	30280	85	314	328	165
0	0	0	32	4	2	12355	30275	70	389	409	244
0	0	0	32	5	2	12730	30275	85	312	326	173
0	0	0	32	6	2	13110	30255	75	361	392	168
0	0	0	32	1	3	11210	30640	75	460	468	169
0	0	0	32	2	3	11605	30645	85	318	326	150
0	0	0	32	3	3	11995	30650	90	215	219	116
0	0	0	32	4	3	12350	30660	95	251	264	158
0	0	0	32	5	3	12715	30650	90	271	290	173
0	0	0	32	6	3	13105	30640	70	380	395	139
0	0	0	32	1	4	11215	31025	80	424	439	213
0	0	0	32	2	4	11605	31020	80	322	336	161
0	0	0	32	3	4	11995	31015	95	210	219	111
0	0	0	32	4	4	12350	31005	95	258	275	158
0	0	0	32	5	4	12715	31020	85	321	332	190
0	0	0	32	6	4	13105	31030	80	298	341	328
0	0	0	32	1	5	11210	31410	80	447	440	192
0	0	0	32	2	5	11595	31405	80	353	371	187
0	0	0	32	3	5	11975	31395	70	349	368	135
0	0	0	32	4	5	12355	31390	85	342	358	187
0	0	0	32	5	5	12725	31395	95	283	290	160
0	0	0	32	6	5	13110	31415	85	298	316	158
0	0	0	32	1	6	11195	31790	75	492	518	214
0	0	0	32	2	6	11580	31775	75	485	496	168
0	0	0	32	3	6	11970	31770	70	508	519	207
0	0	0	32	4	6	12360	31775	75	433	467	201
0	0	0	32	5	6	12745	31775	80	313	349	383
0	0	0	32	6	6	13125	31785	75	366	385	149
0	0	0	33	1	1	11215	33785	70	77	78	35
0	0	0	33	2	1	11595	33785	55	90	104	66
0	0	0	33	3	1	11975	33800	50	86	95	62
0	0	0	33	4	1	12360	33800	55	90	94	31
0	0	0	33	5	1	12740	33800	65	84	87	34
0	0	0	33	6	1	13115	33780	55	83	103	80
0	0	0	33	1	2	11225	34150	55	92	94	31
0	0	0	33	2	2	11620	34170	60	93	111	104
0	0	0	33	3	2	11995	34185	55	90	94	40
0	0	0	33	4	2	12360	34190	65	80	82	34
0	0	0	33	5	2	12730	34185	60	72	80	35
0	0	0	33	6	2	13100	34160	55	73	79	29
0	0	0	33	1	3	11230	34535	60	82	94	43
0	0	0	33	2	3	11625	34540	65	91	96	45
0	0	0	33	3	3	12010	34555	45	76	82	34

0	0	0	33	4	3	12350	34570	55	76	80	29
0	0	0	33	5	3	12715	34545	50	85	92	40
0	0	0	33	6	3	13100	34545	60	72	75	33
0	0	0	33	1	4	11235	34915	60	76	86	41
0	0	0	33	2	4	11625	34915	55	80	84	34
0	0	0	33	3	4	12015	34900	55	83	87	34
0	0	0	33	4	4	12360	34885	50	84	92	38
0	0	0	33	5	4	12710	34910	60	82	90	43
0	0	0	33	6	4	13100	34915	50	80	81	34
0	0	0	33	1	5	11225	35305	60	78	83	29
0	0	0	33	2	5	11615	35290	60	85	100	78
0	0	0	33	3	5	11990	35275	60	77	80	32
0	0	0	33	4	5	12360	35270	50	82	85	30
0	0	0	33	5	5	12725	35285	65	76	80	32
0	0	0	33	6	5	13110	35315	45	85	92	44
0	0	0	33	1	6	11215	35675	70	75	82	35
0	0	0	33	2	6	11605	35670	50	81	85	34
0	0	0	33	3	6	11980	35660	60	90	91	39
0	0	0	33	4	6	12365	35665	60	80	88	38
0	0	0	33	5	6	12740	35665	50	88	89	38
0	0	0	33	6	6	13125	35685	40	86	92	37
0	0	0	34	1	1	11200	37670	85	11645	11991	3441
0	0	0	34	2	1	11580	37685	85	8851	9067	1864
0	0	0	34	3	1	11965	37690	85	7716	7928	1668
0	0	0	34	4	1	12360	37695	85	7083	7316	1653
0	0	0	34	5	1	12735	37690	90	6554	6580	1987
0	0	0	34	6	1	13110	37675	90	6734	6541	2431
0	0	0	34	1	2	11210	38050	85	9582	9696	2002
0	0	0	34	2	2	11600	38065	95	7349	7583	1641
0	0	0	34	3	2	11975	38080	95	5672	5805	1337
0	0	0	34	4	2	12350	38085	95	5474	5422	1491
0	0	0	34	5	2	12715	38070	95	5261	5270	1342
0	0	0	34	6	2	13095	38055	90	5873	5747	1759
0	0	0	34	1	3	11215	38430	85	9955	10155	2122
0	0	0	34	2	3	11610	38440	95	8468	8461	1585
0	0	0	34	3	3	12000	38450	105	5856	6360	5057
0	0	0	34	4	3	12340	38470	105	5115	4793	1555
0	0	0	34	5	3	12700	38445	100	4925	4638	1533
0	0	0	34	6	3	13090	38435	90	5677	5567	1562
0	0	0	34	1	4	11215	38820	90	14780	14085	5209
0	0	0	34	2	4	11610	38815	100	13257	12643	5190
0	0	0	34	3	4	11995	38800	105	8420	8098	2927
0	0	0	34	4	4	12345	38790	105	6146	5755	1925
0	0	0	34	5	4	12705	38810	100	5767	5405	1831
0	0	0	34	6	4	13090	38820	90	6111	6055	1844
0	0	0	34	1	5	11210	39205	95	29370	10048	11558
0	0	0	34	2	5	11600	39195	95	30417	10822	8676
0	0	0	34	3	5	11975	39180	95	16063	17583	6159

0	0	0	34	4	5	12355	39180	95	9059	9292	2358
0	0	0	34	5	5	12720	39190	95	7057	7094	1392
0	0	0	34	6	5	13100	39210	90	6813	6740	1861
0	0	0	34	1	6	11195	39580	105	20598	12244	24363
0	0	0	34	2	6	11580	39575	105	20184	11581	24658
0	0	0	34	3	6	11965	39570	85	24363	15415	7144
0	0	0	34	4	6	12360	39570	90	11448	11896	5675
0	0	0	34	5	6	12740	39570	85	8026	8104	1413
0	0	0	34	6	6	13115	39585	85	7567	7583	1200
0	0	0	35	1	1	11200	41570	65	438	445	163
0	0	0	35	2	1	11580	41585	65	397	415	150
0	0	0	35	3	1	11965	41590	65	394	411	167
0	0	0	35	4	1	12355	41590	70	363	358	183
0	0	0	35	5	1	12735	41585	65	385	393	156
0	0	0	35	6	1	13115	41575	65	445	459	252
0	0	0	35	1	2	11210	41955	60	456	522	670
0	0	0	35	2	2	11595	41965	65	313	335	125
0	0	0	35	3	2	11975	41975	60	377	392	153
0	0	0	35	4	2	12350	41980	65	329	337	139
0	0	0	35	5	2	12720	41970	60	305	312	118
0	0	0	35	6	2	13100	41955	60	387	410	143
0	0	0	35	1	3	11215	42335	70	329	331	152
0	0	0	35	2	3	11605	42340	65	324	320	114
0	0	0	35	3	3	11990	42345	65	303	317	132
0	0	0	35	4	3	12350	42355	70	253	266	134
0	0	0	35	5	3	12710	42345	65	329	332	127
0	0	0	35	6	3	13095	42340	65	472	459	161
0	0	0	35	1	4	11215	42725	65	389	403	144
0	0	0	35	2	4	11605	42720	75	268	285	164
0	0	0	35	3	4	11990	42715	65	307	331	143
0	0	0	35	4	4	12350	42700	75	260	261	112
0	0	0	35	5	4	12715	42715	65	263	270	109
0	0	0	35	6	4	13095	42725	65	469	522	229
0	0	0	35	1	5	11210	43105	65	389	417	149
0	0	0	35	2	5	11595	43095	65	436	442	164
0	0	0	35	3	5	11975	43090	65	360	391	218
0	0	0	35	4	5	12350	43085	60	296	302	114
0	0	0	35	5	5	12725	43095	60	274	298	111
0	0	0	35	6	5	13100	43110	65	357	351	137
0	0	0	35	1	6	11195	43485	70	368	411	332
0	0	0	35	2	6	11585	43475	65	403	410	151
0	0	0	35	3	6	11970	43470	70	301	318	156
0	0	0	35	4	6	12355	43470	70	313	321	176
0	0	0	35	5	6	12740	43475	60	398	421	167
0	0	0	35	6	6	13115	43490	65	464	502	208
0	0	0	36	1	1	11195	45470	95	1001	1090	571
0	0	0	36	2	1	11580	45480	85	972	977	373
0	0	0	36	3	1	11965	45490	90	795	855	372

0	0	0	36	4	1	12355	45490	90	780	819	369
0	0	0	36	5	1	12735	45485	90	836	828	360
0	0	0	36	6	1	13115	45475	95	750	757	376
0	0	0	36	1	2	11210	45850	90	856	943	477
0	0	0	36	2	2	11595	45865	100	665	705	423
0	0	0	36	3	2	11975	45880	100	673	714	415
0	0	0	36	4	2	12350	45880	95	641	675	300
0	0	0	36	5	2	12715	45880	100	504	551	296
0	0	0	36	6	2	13100	45855	90	706	741	340
0	0	0	36	1	3	11210	46235	90	1016	1018	414
0	0	0	36	2	3	11605	46245	100	664	680	384
0	0	0	36	3	3	11995	46255	110	391	496	314
0	0	0	36	4	3	12340	46270	115	402	497	291
0	0	0	36	5	3	12705	46245	105	461	505	278
0	0	0	36	6	3	13100	46235	85	670	1059	3450
0	0	0	36	1	4	11210	46630	90	730	774	347
0	0	0	36	2	4	11600	46620	95	605	645	284
0	0	0	36	3	4	11995	46610	110	371	447	267
0	0	0	36	4	4	12345	46595	110	463	474	208
0	0	0	36	5	4	12710	46620	105	445	501	282
0	0	0	36	6	4	13100	46625	85	719	760	293
0	0	0	36	1	5	11200	47010	85	673	697	312
0	0	0	36	2	5	11595	47005	95	550	569	270
0	0	0	36	3	5	11970	46990	95	562	577	218
0	0	0	36	4	5	12355	46990	90	532	561	239
0	0	0	36	5	5	12720	46995	105	503	556	302
0	0	0	36	6	5	13105	47015	85	740	768	263
0	0	0	36	1	6	11195	47395	95	572	621	284
0	0	0	36	2	6	11580	47375	90	269	311	270
0	0	0	36	3	6	11965	47370	90	614	647	307
0	0	0	36	4	6	12350	47375	80	627	677	297
0	0	0	36	5	6	12740	47380	80	721	760	274
0	0	0	36	6	6	13115	47390	95	761	753	331
0	0	0	37	1	1	11195	49370	95	587	630	325
0	0	0	37	2	1	11580	49385	90	460	491	207
0	0	0	37	3	1	11965	49385	95	537	581	293
0	0	0	37	4	1	12350	49390	90	591	631	274
0	0	0	37	5	1	12735	49385	95	607	701	397
0	0	0	37	6	1	13110	49370	95	564	649	343
0	0	0	37	1	2	11205	49750	90	437	472	271
0	0	0	37	2	2	11590	49770	100	431	445	303
0	0	0	37	3	2	11975	49780	105	364	407	231
0	0	0	37	4	2	12350	49785	100	380	460	277
0	0	0	37	5	2	12720	49775	105	418	495	287
0	0	0	37	6	2	13100	49755	90	601	638	375
0	0	0	37	1	3	11210	50130	90	463	505	239
0	0	0	37	2	3	11600	50140	105	393	402	179
0	0	0	37	3	3	11990	50155	115	285	350	206

0	0	0	37	4	3	12340	50175	120	376	399	212
0	0	0	37	5	3	12705	50150	105	518	514	247
0	0	0	37	6	3	13095	50140	95	647	678	300
0	0	0	37	1	4	11205	50525	90	563	653	410
0	0	0	37	2	4	11600	50520	100	388	385	151
0	0	0	37	3	4	11985	50500	115	322	340	167
0	0	0	37	4	4	12345	50490	110	430	485	272
0	0	0	37	5	4	12705	50510	110	454	479	308
0	0	0	37	6	4	13100	50525	95	742	811	377
0	0	0	37	1	5	11205	50915	95	528	567	263
0	0	0	37	2	5	11590	50905	100	416	442	399
0	0	0	37	3	5	11975	50895	105	437	450	243
0	0	0	37	4	5	12345	50890	105	475	486	257
0	0	0	37	5	5	12720	50900	105	595	652	913
0	0	0	37	6	5	13105	50915	95	716	762	379
0	0	0	37	1	6	11190	51290	90	509	531	193
0	0	0	37	2	6	11580	51285	85	483	520	204
0	0	0	37	3	6	11965	51280	90	477	558	295
0	0	0	37	4	6	12355	51280	95	577	627	286
0	0	0	37	5	6	12740	51280	95	684	788	516
0	0	0	37	6	6	13120	51290	95	664	758	396
0	0	0	38	1	1	11195	53270	80	330	362	179
0	0	0	38	2	1	11580	53285	80	364	385	180
0	0	0	38	3	1	11970	53290	80	292	324	168
0	0	0	38	4	1	12355	53290	75	536	615	332
0	0	0	38	5	1	12745	53290	75	497	539	221
0	0	0	38	6	1	13120	53275	85	500	538	292
0	0	0	38	1	2	11210	53650	80	296	322	163
0	0	0	38	2	2	11595	53665	80	249	275	132
0	0	0	38	3	2	11980	53670	75	238	256	115
0	0	0	38	4	2	12355	53680	85	354	368	228
0	0	0	38	5	2	12730	53670	80	386	414	245
0	0	0	38	6	2	13105	53660	80	391	430	234
0	0	0	38	1	3	11210	54035	75	325	349	175
0	0	0	38	2	3	11600	54035	80	222	241	134
0	0	0	38	3	3	12000	54050	90	183	182	94
0	0	0	38	4	3	12340	54060	75	372	381	137
0	0	0	38	5	3	12715	54040	75	354	360	137
0	0	0	38	6	3	13100	54035	75	401	441	222
0	0	0	38	1	4	11210	54425	75	346	364	192
0	0	0	38	2	4	11600	54420	75	244	259	94
0	0	0	38	3	4	11995	54410	90	182	188	97
0	0	0	38	4	4	12350	54400	90	222	256	319
0	0	0	38	5	4	12720	54420	75	373	383	167
0	0	0	38	6	4	13105	54430	75	400	416	166
0	0	0	38	1	5	11210	54810	80	276	291	127
0	0	0	38	2	5	11595	54805	80	222	229	116
0	0	0	38	3	5	11970	54790	80	254	273	188

0	0	0	38	4	5	12355	54790	70	325	353	270
0	0	0	38	5	5	12730	54800	80	311	333	185
0	0	0	38	6	5	13110	54815	75	389	414	227
0	0	0	38	1	6	11200	55185	80	292	309	131
0	0	0	38	2	6	11580	55180	75	264	278	111
0	0	0	38	3	6	11965	55175	75	303	332	155
0	0	0	38	4	6	12360	55175	80	314	350	175
0	0	0	38	5	6	12745	55180	75	426	445	204
0	0	0	38	6	6	13120	55195	80	408	425	232
0	0	0	39	1	1	11190	57170	85	624	659	231
0	0	0	39	2	1	11575	57185	85	480	513	191
0	0	0	39	3	1	11960	57190	85	638	694	429
0	0	0	39	4	1	12345	57190	80	412	448	220
0	0	0	39	5	1	12730	57185	85	527	542	237
0	0	0	39	6	1	13105	57175	85	633	692	297
0	0	0	39	1	2	11200	57555	85	616	667	349
0	0	0	39	2	2	11590	57565	90	477	491	261
0	0	0	39	3	2	11965	57580	90	442	465	249
0	0	0	39	4	2	12345	57585	90	450	469	280
0	0	0	39	5	2	12715	57575	95	452	480	290
0	0	0	39	6	2	13095	57550	85	592	600	277
0	0	0	39	1	3	11205	57935	85	590	622	229
0	0	0	39	2	3	11595	57940	80	497	508	210
0	0	0	39	3	3	11990	57955	105	358	359	200
0	0	0	39	4	3	12330	57965	105	317	313	178
0	0	0	39	5	3	12705	57940	80	412	425	180
0	0	0	39	6	3	13085	57935	85	536	583	268
0	0	0	39	1	4	11205	58320	85	616	647	257
0	0	0	39	2	4	11600	58310	90	432	456	237
0	0	0	39	3	4	11985	58300	100	381	378	225
0	0	0	39	4	4	12340	58290	105	327	371	661
0	0	0	39	5	4	12700	58315	95	382	391	233
0	0	0	39	6	4	13085	58320	85	583	598	255
0	0	0	39	1	5	11205	58705	85	598	658	269
0	0	0	39	2	5	11590	58700	95	453	478	232
0	0	0	39	3	5	11970	58680	90	407	435	232
0	0	0	39	4	5	12345	58685	85	486	512	231
0	0	0	39	5	5	12715	58690	95	466	489	254
0	0	0	39	6	5	13095	58705	85	593	637	262
0	0	0	39	1	6	11190	59085	80	726	843	1040
0	0	0	39	2	6	11580	59075	80	529	558	200
0	0	0	39	3	6	11960	59070	80	557	627	304
0	0	0	39	4	6	12350	59070	85	538	571	222
0	0	0	39	5	6	12730	59075	80	585	643	441
0	0	0	39	6	6	13110	59090	85	602	621	275
0	0	0	40	1	1	11210	61065	140	1528	3297	3343
0	0	0	40	2	1	11600	61075	135	1551	3231	3108
0	0	0	40	3	1	11980	61080	135	1342	3137	3024

0	0	0	40	4	1	12370	61085	140	1361	2653	2560
0	0	0	40	5	1	12755	61080	140	1439	2778	2618
0	0	0	40	6	1	13130	61065	135	1446	3028	2911
0	0	0	40	1	2	11225	61450	140	1264	2390	2329
0	0	0	40	2	2	11610	61460	145	1491	2996	2772
0	0	0	40	3	2	11985	61475	145	1925	2895	2609
0	0	0	40	4	2	12365	61480	145	1307	2319	2018
0	0	0	40	5	2	12735	61470	145	1448	2552	2316
0	0	0	40	6	2	13120	61455	140	1190	2480	2417
0	0	0	40	1	3	11225	61825	135	1320	2640	2454
0	0	0	40	2	3	11625	61835	145	1302	2209	1963
0	0	0	40	3	3	12010	61850	150	1804	2371	2022
0	0	0	40	4	3	12355	61860	150	1734	2140	1767
0	0	0	40	5	3	12725	61840	150	1678	2352	2073
0	0	0	40	6	3	13115	61830	140	1221	2348	2169
0	0	0	40	1	4	11225	62215	135	1756	3238	3255
0	0	0	40	2	4	11620	62215	145	1968	2976	2491
0	0	0	40	3	4	12010	62205	150	1689	2423	2110
0	0	0	40	4	4	12360	62195	155	1845	2351	2053
0	0	0	40	5	4	12720	62220	150	1648	2519	2285
0	0	0	40	6	4	13110	62225	135	1408	2643	2486
0	0	0	40	1	5	11220	62605	145	2620	4938	4645
0	0	0	40	2	5	11615	62595	145	2566	4163	3457
0	0	0	40	3	5	11990	62585	145	2960	4265	3445
0	0	0	40	4	5	12370	62580	145	2822	4151	3400
0	0	0	40	5	5	12740	62585	145	2258	3686	3049
0	0	0	40	6	5	13115	62610	135	2031	3652	3658
0	0	0	40	1	6	11210	62990	140	1940	3900	3865
0	0	0	40	2	6	11595	62975	145	1855	3759	3600
0	0	0	40	3	6	11980	62975	140	1837	3683	3815
0	0	0	40	4	6	12370	62965	140	1709	3183	2954
0	0	0	40	5	6	12750	62970	135	1781	3293	3173
0	0	0	40	6	6	13130	62980	135	1806	2865	2631
0	0	0	41	1	1	15095	14275	105	7434	7514	1649
0	0	0	41	2	1	15480	14290	105	6379	6626	2371
0	0	0	41	3	1	15865	14295	100	6102	6237	1256
0	0	0	41	4	1	16255	14295	100	6642	6708	992
0	0	0	41	5	1	16640	14290	100	6759	6907	1176
0	0	0	41	6	1	17020	14280	105	7263	7279	1338
0	0	0	41	1	2	15110	14655	105	7170	7275	1624
0	0	0	41	2	2	15500	14670	110	6266	6208	1750
0	0	0	41	3	2	15875	14685	110	5994	6100	1677
0	0	0	41	4	2	16255	14690	110	6397	6435	1290
0	0	0	41	5	2	16625	14680	115	6218	6282	1473
0	0	0	41	6	2	17005	14660	110	6885	6304	2444
0	0	0	41	1	3	15110	15045	105	7212	7412	1530
0	0	0	41	2	3	15510	15050	110	6969	7029	1707
0	0	0	41	3	3	15895	15060	120	6580	6566	1889

0	0	0	41	4	3	16250	15075	120	6208	6335	2770
0	0	0	41	5	3	16610	15050	115	6087	6308	2219
0	0	0	41	6	3	17000	15045	105	6927	7095	1264
0	0	0	41	1	4	15110	15430	105	7688	7803	1410
0	0	0	41	2	4	15505	15425	110	21310	30900	20174
0	0	0	41	3	4	15895	15415	120	5964	5930	1713
0	0	0	41	4	4	16245	15400	115	5721	5932	972
0	0	0	41	5	4	16610	15425	115	5971	6158	1141
0	0	0	41	6	4	17000	15430	105	6879	7029	966
0	0	0	41	1	5	15105	15815	105	8741	8870	1555
0	0	0	41	2	5	15500	15810	110	7758	7582	2656
0	0	0	41	3	5	15875	15795	110	7157	7082	1453
0	0	0	41	4	5	16255	15790	110	6899	6968	1561
0	0	0	41	5	5	16625	15800	110	7116	7108	1439
0	0	0	41	6	5	17005	15820	105	7028	7406	2778
0	0	0	41	1	6	15095	16200	105	8182	8451	2420
0	0	0	41	2	6	15480	16180	105	8496	8445	1533
0	0	0	41	3	6	15870	16175	100	8316	8358	2201
0	0	0	41	4	6	16255	16180	100	9026	9943	5996
0	0	0	41	5	6	16640	16180	105	8269	8455	1605
0	0	0	41	6	6	17020	16195	105	7623	7920	1670
0	0	0	42	1	1	15095	18175	95	3333	3379	1082
0	0	0	42	2	1	15480	18190	100	3436	3105	1114
0	0	0	42	3	1	15865	18195	100	3734	3549	1167
0	0	0	42	4	1	16250	18195	100	4332	4213	1990
0	0	0	42	5	1	16635	18195	100	3723	3608	1174
0	0	0	42	6	1	17010	18180	100	3364	3228	1017
0	0	0	42	1	2	15110	18560	100	2868	2770	904
0	0	0	42	2	2	15500	18570	110	3382	3046	1260
0	0	0	42	3	2	15875	18585	105	3154	3060	820
0	0	0	42	4	2	16250	18590	110	3438	3286	1247
0	0	0	42	5	2	16620	18580	110	3369	3091	1244
0	0	0	42	6	2	17000	18560	105	3263	3164	961
0	0	0	42	1	3	15110	18950	90	4776	5338	2649
0	0	0	42	2	3	15505	18945	105	3345	3306	879
0	0	0	42	3	3	15895	18960	115	2419	2397	689
0	0	0	42	4	3	16235	18975	115	2835	2890	950
0	0	0	42	5	3	16600	18950	115	3037	3043	1746
0	0	0	42	6	3	16990	18945	105	3271	3264	1179
0	0	0	42	1	4	15110	19325	95	2962	2912	634
0	0	0	42	2	4	15505	19320	105	2849	2793	940
0	0	0	42	3	4	15890	19310	115	2289	2323	886
0	0	0	42	4	4	16245	19295	115	3530	3686	1796
0	0	0	42	5	4	16605	19320	110	3022	2883	956
0	0	0	42	6	4	16995	19330	105	3197	3115	1073
0	0	0	42	1	5	15110	19715	105	2973	2818	867
0	0	0	42	2	5	15495	19700	105	2795	2719	895
0	0	0	42	3	5	15875	19690	110	2842	2691	1100

0	0	0	42	4	5	16255	19685	110	3062	2873	1173
0	0	0	42	5	5	16615	19695	105	3429	3523	1533
0	0	0	42	6	5	17000	19715	105	3265	3219	1118
0	0	0	42	1	6	15095	20090	100	3144	3150	1388
0	0	0	42	2	6	15480	20085	100	3555	3418	1200
0	0	0	42	3	6	15865	20075	95	4009	3911	1264
0	0	0	42	4	6	16250	20075	95	4113	4143	1169
0	0	0	42	5	6	16640	20080	100	3854	3711	1131
0	0	0	42	6	6	17015	20095	100	3890	3864	1331
0	0	0	43	1	1	15095	22075	105	7319	7111	1681
0	0	0	43	2	1	15480	22085	95	6504	6591	1678
0	0	0	43	3	1	15865	22095	100	5626	5471	1133
0	0	0	43	4	1	16250	22095	95	5880	5869	681
0	0	0	43	5	1	16635	22095	100	7191	8677	7822
0	0	0	43	6	1	17015	22080	100	5955	5857	1356
0	0	0	43	1	2	15110	22455	105	7095	7006	2343
0	0	0	43	2	2	15500	22470	110	5888	5675	2205
0	0	0	43	3	2	15875	22485	110	5204	5090	2258
0	0	0	43	4	2	16250	22490	110	5873	5657	1358
0	0	0	43	5	2	16620	22480	110	6521	8024	8310
0	0	0	43	6	2	17000	22460	100	5696	5656	1143
0	0	0	43	1	3	15110	22840	100	6827	6632	1680
0	0	0	43	2	3	15510	22850	110	5829	5544	1763
0	0	0	43	3	3	15895	22860	115	5384	5547	2170
0	0	0	43	4	3	16240	22875	120	4977	4748	1392
0	0	0	43	5	3	16605	22850	110	4968	4960	1273
0	0	0	43	6	3	16995	22845	100	5304	5256	1224
0	0	0	43	1	4	15110	23230	100	7459	7320	1741
0	0	0	43	2	4	15505	23225	105	6440	7364	4756
0	0	0	43	3	4	15895	23215	115	5502	5611	1422
0	0	0	43	4	4	16245	23200	115	5405	5535	1283
0	0	0	43	5	4	16605	23220	110	5350	5317	1324
0	0	0	43	6	4	16995	23230	100	5770	5879	1646
0	0	0	43	1	5	15110	23615	100	8535	8422	1982
0	0	0	43	2	5	15500	23605	105	6085	6136	914
0	0	0	43	3	5	15880	23590	105	5295	5384	987
0	0	0	43	4	5	16255	23585	105	5933	6058	1016
0	0	0	43	5	5	16625	23595	105	6084	6176	1197
0	0	0	43	6	5	17000	23615	100	6234	6338	1672
0	0	0	43	1	6	15095	23990	105	11832	12073	3441
0	0	0	43	2	6	15485	23985	100	7165	7113	1812
0	0	0	43	3	6	15865	23980	100	6170	6091	1833
0	0	0	43	4	6	16260	23975	95	6865	7099	1592
0	0	0	43	5	6	16640	23980	95	7325	7525	1348
0	0	0	43	6	6	17015	23995	100	7167	7310	2185
0	0	0	44	1	1	15100	25975	90	1051	1274	989
0	0	0	44	2	1	15485	25985	90	960	1260	922
0	0	0	44	3	1	15870	25990	90	895	1095	714

0	0	0	44	4	1	16255	25995	90	857	1049	769
0	0	0	44	5	1	16635	25990	90	712	950	684
0	0	0	44	6	1	17015	25980	95	709	1013	864
0	0	0	44	1	2	15110	26355	90	882	1030	656
0	0	0	44	2	2	15500	26370	100	642	856	630
0	0	0	44	3	2	15880	26385	105	541	741	572
0	0	0	44	4	2	16255	26390	105	590	731	530
0	0	0	44	5	2	16620	26375	100	507	728	606
0	0	0	44	6	2	17000	26355	90	796	1042	797
0	0	0	44	1	3	15115	26740	90	888	1100	759
0	0	0	44	2	3	15510	26745	100	596	812	636
0	0	0	44	3	3	15900	26755	110	408	552	390
0	0	0	44	4	3	16240	26770	120	305	505	513
0	0	0	44	5	3	16600	26750	110	426	635	562
0	0	0	44	6	3	16995	26745	95	622	927	782
0	0	0	44	1	4	15115	27125	95	1038	1223	794
0	0	0	44	2	4	15510	27120	100	781	914	641
0	0	0	44	3	4	15895	27105	110	401	570	495
0	0	0	44	4	4	16245	27095	110	381	564	482
0	0	0	44	5	4	16605	27115	105	604	713	487
0	0	0	44	6	4	16995	27125	90	897	1133	866
0	0	0	44	1	5	15110	27510	90	994	1145	720
0	0	0	44	2	5	15500	27495	95	775	1017	729
0	0	0	44	3	5	15875	27485	100	596	914	768
0	0	0	44	4	5	16250	27485	100	571	806	621
0	0	0	44	5	5	16620	27495	100	605	863	687
0	0	0	44	6	5	17000	27510	100	774	1008	812
0	0	0	44	1	6	15095	27890	95	1206	1358	993
0	0	0	44	2	6	15485	27885	90	961	1327	1028
0	0	0	44	3	6	15870	27880	90	1077	1314	935
0	0	0	44	4	6	16255	27875	85	808	1063	763
0	0	0	44	5	6	16640	27880	90	869	1174	857
0	0	0	44	6	6	17015	27890	90	1070	1461	1255
0	0	0	45	1	1	15100	29880	105	2433	2619	1457
0	0	0	45	2	1	15480	29895	100	2410	2459	1306
0	0	0	45	3	1	15865	29900	100	2460	2577	1454
0	0	0	45	4	1	16255	29900	105	2126	2176	1131
0	0	0	45	5	1	16635	29895	100	1904	2015	1021
0	0	0	45	6	1	17015	29880	105	1589	1684	858
0	0	0	45	1	2	15110	30260	100	2263	2385	1281
0	0	0	45	2	2	15500	30275	110	1976	2393	2068
0	0	0	45	3	2	15875	30290	110	1489	1825	1145
0	0	0	45	4	2	16250	30295	110	1126	1449	877
0	0	0	45	5	2	16620	30280	115	1161	1448	917
0	0	0	45	6	2	17000	30260	105	1704	1796	1100
0	0	0	45	1	3	15115	30650	100	2173	2465	1374
0	0	0	45	2	3	15510	30650	110	1781	2112	1273
0	0	0	45	3	3	15895	30660	115	721	929	578

0	0	0	45	4	3	16240	30680	120	868	1167	843
0	0	0	45	5	3	16605	30655	120	722	986	730
0	0	0	45	6	3	16995	30650	100	982	1244	894
0	0	0	45	1	4	15095	31015	100	1719	1905	1062
0	0	0	45	2	4	15505	31025	110	1437	1809	1283
0	0	0	45	3	4	15895	31015	120	845	1135	753
0	0	0	45	4	4	16245	31000	120	750	975	639
0	0	0	45	5	4	16605	31025	115	812	1027	603
0	0	0	45	6	4	17000	31030	105	1119	1258	683
0	0	0	45	1	5	15110	31415	100	1598	1891	1247
0	0	0	45	2	5	15500	31405	110	1353	1559	1018
0	0	0	45	3	5	15875	31390	110	931	1106	646
0	0	0	45	4	5	16255	31390	110	942	1128	662
0	0	0	45	5	5	16625	31395	115	808	958	522
0	0	0	45	6	5	17000	31420	105	1031	1131	610
0	0	0	45	1	6	15095	31795	100	1610	1767	872
0	0	0	45	2	6	15480	31785	100	2043	2175	1118
0	0	0	45	3	6	15870	31775	100	1117	1173	586
0	0	0	45	4	6	16255	31775	100	1410	1446	688
0	0	0	45	5	6	16640	31780	100	983	1043	511
0	0	0	45	6	6	17015	31795	105	1176	1219	610
0	0	0	46	1	1	15095	33775	90	267	290	173
0	0	0	46	2	1	15480	33785	85	445	465	254
0	0	0	46	3	1	15865	33790	90	338	397	252
0	0	0	46	4	1	16255	33785	80	308	361	223
0	0	0	46	5	1	16640	33785	85	369	404	211
0	0	0	46	6	1	17020	33775	90	240	275	167
0	0	0	46	1	2	15105	34155	85	384	438	236
0	0	0	46	2	2	15500	34170	100	311	398	297
0	0	0	46	3	2	15875	34180	95	247	312	209
0	0	0	46	4	2	16255	34185	100	179	231	160
0	0	0	46	5	2	16625	34175	105	185	232	155
0	0	0	46	6	2	17005	34160	90	191	231	138
0	0	0	46	1	3	15105	34540	80	332	383	199
0	0	0	46	2	3	15505	34545	95	252	311	204
0	0	0	46	3	3	15895	34550	105	178	229	162
0	0	0	46	4	3	16255	34565	95	155	190	121
0	0	0	46	5	3	16620	34545	80	213	240	139
0	0	0	46	6	3	17010	34540	70	248	277	140
0	0	0	46	1	4	15110	34935	80	295	326	174
0	0	0	46	2	4	15505	34920	90	236	277	179
0	0	0	46	3	4	15895	34910	110	147	193	143
0	0	0	46	4	4	16245	34895	115	134	171	114
0	0	0	46	5	4	16605	34920	110	129	168	121
0	0	0	46	6	4	17005	34930	95	205	238	150
0	0	0	46	1	5	15105	35315	90	246	280	168
0	0	0	46	2	5	15495	35305	95	241	290	181
0	0	0	46	3	5	15875	35290	100	186	234	156

0	0	0	46	4	5	16255	35300	80	209	245	145
0	0	0	46	5	5	16625	35295	100	151	180	107
0	0	0	46	6	5	17005	35320	90	197	225	129
0	0	0	46	1	6	15095	35695	90	288	307	184
0	0	0	46	2	6	15480	35690	85	292	326	180
0	0	0	46	3	6	15865	35685	80	297	324	188
0	0	0	46	4	6	16255	35685	80	267	293	147
0	0	0	46	5	6	16640	35690	80	227	245	123
0	0	0	46	6	6	17025	35700	85	265	270	129
0	0	0	47	1	1	15090	37670	105	165	177	77
0	0	0	47	2	1	15470	37680	105	161	177	89
0	0	0	47	3	1	15860	37695	110	154	168	81
0	0	0	47	4	1	16250	37695	110	219	231	117
0	0	0	47	5	1	16630	37690	110	141	154	86
0	0	0	47	6	1	17020	37670	105	144	151	69
0	0	0	47	1	2	15100	38055	100	155	163	77
0	0	0	47	2	2	15490	38070	115	132	147	95
0	0	0	47	3	2	15870	38080	120	137	154	85
0	0	0	47	4	2	16245	38085	115	136	162	155
0	0	0	47	5	2	16615	38075	115	120	130	59
0	0	0	47	6	2	17000	38055	105	122	138	67
0	0	0	47	1	3	15105	38440	105	153	173	99
0	0	0	47	2	3	15495	38445	105	139	158	109
0	0	0	47	3	3	15925	38490	30	90	97	40
0	0	0	47	4	3	16250	38465	105	124	140	77
0	0	0	47	5	3	16605	38450	115	121	130	60
0	0	0	47	6	3	17010	38440	85	145	149	61
0	0	0	47	1	4	15100	38830	90	172	188	88
0	0	0	47	2	4	15500	38825	115	117	128	61
0	0	0	47	3	4	15885	38815	115	116	127	58
0	0	0	47	4	4	16240	38800	120	110	125	74
0	0	0	47	5	4	16605	38820	120	104	118	59
0	0	0	47	6	4	17000	38830	105	130	144	66
0	0	0	47	1	5	15100	39220	105	160	177	93
0	0	0	47	2	5	15490	39205	110	153	166	76
0	0	0	47	3	5	15870	39195	115	154	185	346
0	0	0	47	4	5	16245	39190	115	138	156	81
0	0	0	47	5	5	16620	39205	115	122	129	66
0	0	0	47	6	5	17000	39220	110	103	113	55
0	0	0	47	1	6	15090	39595	105	181	192	86
0	0	0	47	2	6	15475	39590	105	173	183	81
0	0	0	47	3	6	15860	39585	100	169	187	92
0	0	0	47	4	6	16250	39590	90	191	204	89
0	0	0	47	5	6	16640	39585	100	153	169	83
0	0	0	47	6	6	17020	39600	105	127	137	64
0	0	0	48	1	1	15090	41575	105	4902	4953	990
0	0	0	48	2	1	15480	41585	105	4576	4656	992
0	0	0	48	3	1	15860	41590	110	4681	4489	1638

0	0	0	48	4	1	16250	41590	110	4512	4319	1415
0	0	0	48	5	1	16635	41585	105	4164	4367	914
0	0	0	48	6	1	17015	41575	110	3499	3329	1084
0	0	0	48	1	2	15105	41955	110	4722	4691	1684
0	0	0	48	2	2	15495	41965	115	4645	4702	1623
0	0	0	48	3	2	15875	41980	115	4236	4358	1124
0	0	0	48	4	2	16250	41985	115	3866	3926	907
0	0	0	48	5	2	16620	41975	115	3704	3664	791
0	0	0	48	6	2	17000	41955	110	2917	2865	902
0	0	0	48	1	3	15105	42340	105	4435	4681	1221
0	0	0	48	2	3	15500	42345	115	4200	4282	1176
0	0	0	48	3	3	15890	42355	125	4134	4242	1290
0	0	0	48	4	3	16240	42370	130	3471	3376	1272
0	0	0	48	5	3	16605	42345	120	3424	3363	993
0	0	0	48	6	3	16995	42340	110	3065	3050	1100
0	0	0	48	1	4	15105	42725	105	4439	4708	1203
0	0	0	48	2	4	15500	42720	115	4059	4193	1089
0	0	0	48	3	4	15890	42710	125	3975	4054	1087
0	0	0	48	4	4	16240	42695	125	3598	3656	888
0	0	0	48	5	4	16605	42720	120	3725	3741	1801
0	0	0	48	6	4	16995	42730	110	3402	3336	1101
0	0	0	48	1	5	15105	43115	110	4549	4556	1763
0	0	0	48	2	5	15490	43100	115	4436	4533	1282
0	0	0	48	3	5	15870	43090	110	4377	4447	1101
0	0	0	48	4	5	16250	43085	115	3927	3960	972
0	0	0	48	5	5	16620	43090	110	3742	3780	869
0	0	0	48	6	5	17000	43115	110	3715	3678	1110
0	0	0	48	1	6	15090	43495	110	4477	4574	1671
0	0	0	48	2	6	15480	43485	110	4520	4377	1703
0	0	0	48	3	6	15860	43480	105	4629	4864	1244
0	0	0	48	4	6	16250	43475	105	4327	4467	1017
0	0	0	48	5	6	16635	43480	105	4219	4366	1027
0	0	0	48	6	6	17015	43495	110	4273	4123	1440
0	0	0	49	1	1	15090	45480	100	664	700	356
0	0	0	49	2	1	15480	45490	100	734	774	397
0	0	0	49	3	1	15865	45495	100	830	892	484
0	0	0	49	4	1	16250	45495	100	815	872	426
0	0	0	49	5	1	16635	45495	100	832	893	497
0	0	0	49	6	1	17010	45480	100	809	790	370
0	0	0	49	1	2	15105	45855	100	642	749	436
0	0	0	49	2	2	15495	45870	105	739	786	421
0	0	0	49	3	2	15875	45885	110	642	685	461
0	0	0	49	4	2	16245	45890	110	460	591	389
0	0	0	49	5	2	16620	45875	105	716	781	425
0	0	0	49	6	2	17000	45860	105	739	797	502
0	0	0	49	1	3	15110	46240	100	708	766	410
0	0	0	49	2	3	15500	46245	105	767	851	491
0	0	0	49	3	3	15870	46255	170	350	520	1372

0	0	0	49	4	3	16235	46275	120	369	470	309
0	0	0	49	5	3	16600	46250	110	464	578	351
0	0	0	49	6	3	16990	46245	105	530	560	281
0	0	0	49	1	4	15110	46630	100	776	830	435
0	0	0	49	2	4	15500	46625	105	663	770	450
0	0	0	49	3	4	15890	46605	120	379	542	423
0	0	0	49	4	4	16240	46600	115	439	556	355
0	0	0	49	5	4	16600	46620	115	469	534	325
0	0	0	49	6	4	16995	46630	105	693	734	418
0	0	0	49	1	5	15105	47015	100	812	886	576
0	0	0	49	2	5	15495	47005	110	506	626	418
0	0	0	49	3	5	15870	46995	105	704	733	432
0	0	0	49	4	5	16250	46990	105	574	646	346
0	0	0	49	5	5	16620	47000	110	441	521	324
0	0	0	49	6	5	17000	47015	105	571	599	293
0	0	0	49	1	6	15090	47400	105	752	831	457
0	0	0	49	2	6	15480	47380	100	652	696	364
0	0	0	49	3	6	15865	47380	95	796	843	376
0	0	0	49	4	6	16250	47375	100	600	650	328
0	0	0	49	5	6	16635	47380	100	568	617	311
0	0	0	49	6	6	17015	47395	105	541	580	351
0	0	0	50	1	1	15085	49375	95	977	966	405
0	0	0	50	2	1	15470	49390	90	813	871	374
0	0	0	50	3	1	15855	49395	90	908	974	474
0	0	0	50	4	1	16250	49395	90	935	1053	676
0	0	0	50	5	1	16630	49390	95	1100	1176	567
0	0	0	50	6	1	17010	49380	100	1062	1139	714
0	0	0	50	1	2	15095	49755	95	912	985	456
0	0	0	50	2	2	15490	49770	100	649	721	451
0	0	0	50	3	2	15865	49785	105	635	756	489
0	0	0	50	4	2	16240	49785	105	731	917	921
0	0	0	50	5	2	16615	49780	105	748	888	569
0	0	0	50	6	2	16995	49760	100	1051	1152	705
0	0	0	50	1	3	15100	50145	90	882	1002	667
0	0	0	50	2	3	15495	50145	105	641	727	443
0	0	0	50	3	3	15885	50160	110	460	664	502
0	0	0	50	4	3	16230	50175	120	362	546	443
0	0	0	50	5	3	16595	50150	105	639	863	633
0	0	0	50	6	3	16990	50140	95	1422	1466	661
0	0	0	50	1	4	15100	50530	90	922	966	464
0	0	0	50	2	4	15495	50525	100	940	1046	635
0	0	0	50	3	4	15885	50515	115	513	746	616
0	0	0	50	4	4	16235	50500	120	460	689	595
0	0	0	50	5	4	16600	50525	110	655	949	790
0	0	0	50	6	4	16990	50530	100	1231	1364	883
0	0	0	50	1	5	15095	50915	90	1037	1083	530
0	0	0	50	2	5	15485	50910	105	863	1013	820
0	0	0	50	3	5	15860	50890	105	762	910	617

0	0	0	50	4	5	16240	50890	105	769	899	593
0	0	0	50	5	5	16615	50900	110	797	960	671
0	0	0	50	6	5	17000	50920	95	1507	1587	731
0	0	0	50	1	6	15085	51295	100	1064	1141	680
0	0	0	50	2	6	15470	51285	90	1169	1206	520
0	0	0	50	3	6	15860	51285	90	1177	1229	531
0	0	0	50	4	6	16245	51285	95	1098	1194	513
0	0	0	50	5	6	16635	51285	90	1339	1434	804
0	0	0	50	6	6	17010	51300	95	1387	1624	2009
0	0	0	51	1	1	15080	53270	120	125	137	96
0	0	0	51	2	1	15470	53275	115	139	147	70
0	0	0	51	3	1	15855	53280	115	131	139	60
0	0	0	51	4	1	16245	53285	110	133	147	68
0	0	0	51	5	1	16640	53285	120	111	118	54
0	0	0	51	6	1	17015	53270	120	119	131	65
0	0	0	51	1	2	15090	53650	120	120	143	237
0	0	0	51	2	2	15480	53660	120	111	126	61
0	0	0	51	3	2	15860	53670	120	104	112	54
0	0	0	51	4	2	16240	53670	110	112	143	343
0	0	0	51	5	2	16640	53660	85	123	139	78
0	0	0	51	6	2	17010	53645	115	108	117	58
0	0	0	51	1	3	15085	54035	100	122	130	59
0	0	0	51	2	3	15485	54040	120	114	126	60
0	0	0	51	3	3	15870	54055	120	99	115	122
0	0	0	51	4	3	16235	54045	95	107	119	54
0	0	0	51	5	3	16625	54040	95	106	112	45
0	0	0	51	6	3	17020	54045	80	129	132	55
0	0	0	51	1	4	15090	54430	110	112	121	74
0	0	0	51	2	4	15525	54435	45	89	90	33
0	0	0	51	3	4	15875	54430	105	102	108	46
0	0	0	51	4	4	16250	54420	100	88	93	39
0	0	0	51	5	4	16620	54435	115	94	122	304
0	0	0	51	6	4	17000	54430	120	94	105	99
0	0	0	51	1	5	15090	54820	110	109	119	53
0	0	0	51	2	5	15475	54815	125	102	108	50
0	0	0	51	3	5	15860	54805	115	91	100	50
0	0	0	51	4	5	16250	54815	95	112	121	52
0	0	0	51	5	5	16630	54815	110	88	99	50
0	0	0	51	6	5	17010	54825	115	96	103	46
0	0	0	51	1	6	15080	55200	120	116	122	52
0	0	0	51	2	6	15460	55200	105	98	104	44
0	0	0	51	3	6	15860	55195	110	94	104	50
0	0	0	51	4	6	16250	55205	95	102	125	203
0	0	0	51	5	6	16640	55200	110	95	148	763
0	0	0	51	6	6	17020	55205	120	92	97	45
0	0	0	52	1	1	15100	57165	110	78	82	40
0	0	0	52	2	1	15485	57175	115	81	87	38
0	0	0	52	3	1	15855	57170	95	87	93	41

0	0	0	52	4	1	16260	57190	120	77	83	40
0	0	0	52	5	1	16655	57170	95	74	80	33
0	0	0	52	6	1	17030	57170	115	82	90	54
0	0	0	52	1	2	15110	57540	95	77	83	34
0	0	0	52	2	2	15535	57580	70	77	81	33
0	0	0	52	3	2	15875	57580	130	84	95	117
0	0	0	52	4	2	16260	57580	130	78	85	35
0	0	0	52	5	2	16635	57555	105	75	81	34
0	0	0	52	6	2	17030	57540	85	72	75	29
0	0	0	52	1	3	15105	57930	100	75	83	36
0	0	0	52	2	3	15490	57945	120	78	82	36
0	0	0	52	3	3	15870	57955	75	80	87	36
0	0	0	52	4	3	16205	57975	65	76	87	88
0	0	0	52	5	3	16595	57925	80	72	76	31
0	0	0	52	6	3	17000	57935	95	81	81	30
0	0	0	52	1	4	15115	58325	110	83	90	61
0	0	0	52	2	4	15515	58325	110	77	92	178
0	0	0	52	3	4	15900	58315	130	85	90	37
0	0	0	52	4	4	16275	58300	95	73	81	34
0	0	0	52	5	4	16640	58310	95	70	77	33
0	0	0	52	6	4	17010	58325	125	73	77	30
0	0	0	52	1	5	15095	58720	95	81	125	603
0	0	0	52	2	5	15465	58685	60	83	87	36
0	0	0	52	3	5	15855	58665	85	69	73	31
0	0	0	52	4	5	16230	58680	80	76	118	531
0	0	0	52	5	5	16595	58675	65	67	73	34
0	0	0	52	6	5	17015	58675	55	71	70	30
0	0	0	52	1	6	15105	59105	110	80	128	816
0	0	0	52	2	6	15460	59100	85	80	109	239
0	0	0	52	3	6	15860	59055	60	75	80	34
0	0	0	52	4	6	16240	59090	55	71	82	48
0	0	0	52	5	6	16620	59060	45	73	78	32
0	0	0	52	6	6	16995	59065	25	81	82	29
0	0	0	53	1	1	15085	61070	95	187	192	76
0	0	0	53	2	1	15475	61085	100	140	155	102
0	0	0	53	3	1	15855	61090	100	119	133	79
0	0	0	53	4	1	16240	61085	90	133	139	63
0	0	0	53	5	1	16630	61090	100	134	143	68
0	0	0	53	6	1	17010	61075	105	140	147	68
0	0	0	53	1	2	15100	61455	95	184	216	261
0	0	0	53	2	2	15485	61465	95	124	136	66
0	0	0	53	3	2	15855	61485	105	106	124	71
0	0	0	53	4	2	16240	61485	100	112	119	55
0	0	0	53	5	2	16620	61470	100	126	131	61
0	0	0	53	6	2	16995	61460	105	123	133	68
0	0	0	53	1	3	15095	61840	85	149	163	133
0	0	0	53	2	3	15495	61845	100	110	118	50
0	0	0	53	3	3	15930	61870	45	56	66	30

0	0	0	53	4	3	16225	61875	120	80	90	50
0	0	0	53	5	3	16605	61845	85	108	121	54
0	0	0	53	6	3	16990	61845	90	139	144	53
0	0	0	53	1	4	15100	62230	95	120	138	110
0	0	0	53	2	4	15490	62230	95	125	132	55
0	0	0	53	3	4	15885	62210	115	90	96	46
0	0	0	53	4	4	16240	62210	85	103	105	50
0	0	0	53	5	4	16600	62230	105	115	128	81
0	0	0	53	6	4	16990	62230	100	117	127	57
0	0	0	53	1	5	15095	62610	90	167	185	95
0	0	0	53	2	5	15485	62620	85	127	134	54
0	0	0	53	3	5	15860	62600	95	147	159	75
0	0	0	53	4	5	16235	62595	95	131	138	53
0	0	0	53	5	5	16620	62600	95	144	162	79
0	0	0	53	6	5	16995	62615	105	163	188	108
0	0	0	53	1	6	15085	63000	95	178	183	57
0	0	0	53	2	6	15470	62985	95	188	197	84
0	0	0	53	3	6	15855	62980	95	177	182	65
0	0	0	53	4	6	16250	62980	85	163	170	61
0	0	0	53	5	6	16630	62980	95	172	183	76
0	0	0	53	6	6	17010	62995	100	166	171	71

00%-pH5.5.tif 0
