# Supplementary material for: pH-dependent and dynamic interactions of cystatin C with heparan sulfate
Source: Commun Biol. 2021 Feb 12;4:198. doi: 10.1038/s42003-021-01737-7 (PMC7881039; doi:10.1038/s42003-021-01737-7)
Supplement: Supplementary file 5 — Supplementary Data 2 [file 42003_2021_1737_MOESM5_ESM.pdf]

papain	papain+Cst-3	papain+Cst-3-heparin complex	papain+Cst-3-H6 complex
2.38	0.15	1.87	1.97
2.23	0.2	1.94	1.89
2.26	0.2	1.73	1.79
1.99	0.24	1.79	1.87
