# Supplementary material for: pH-dependent and dynamic interactions of cystatin C with heparan sulfate
Source: Commun Biol. 2021 Feb 12;4:198. doi: 10.1038/s42003-021-01737-7 (PMC7881039; doi:10.1038/s42003-021-01737-7)
Supplement: Supplementary file 6 — Reporting Summary [file 42003_2021_1737_MOESM6_ESM.pdf]

nature research

Corresponding author(s): Ding Xu

Last updated by author(s): Sep 7, 2020

# Reporting Summary

Nature Research wishes to improve the reproducibility of the work that we publish. This form provides structure for consistency and transparency in reporting. For further information on Nature Research policies, see our Editorial Policies and the Editorial Policy Checklist.

## Statistics

For all statistical analyses, confirm that the following items are present in the figure legend, table legend, main text, or Methods section.

| n/a | Confirmed | |
|---|---|---|
| ☐ | ☒ | The exact sample size (*n*) for each experimental group/condition, given as a discrete number and unit of measurement |
| ☐ | ☒ | A statement on whether measurements were taken from distinct samples or whether the same sample was measured repeatedly |
| ☒ | ☐ | The statistical test(s) used AND whether they are one- or two-sided<br>*Only common tests should be described solely by name; describe more complex techniques in the Methods section.* |
| ☒ | ☐ | A description of all covariates tested |
| ☒ | ☐ | A description of any assumptions or corrections, such as tests of normality and adjustment for multiple comparisons |
| ☒ | ☐ | A full description of the statistical parameters including central tendency (e.g. means) or other basic estimates (e.g. regression coefficient) AND variation (e.g. standard deviation) or associated estimates of uncertainty (e.g. confidence intervals) |
| ☒ | ☐ | For null hypothesis testing, the test statistic (e.g. *F*, *t*, *r*) with confidence intervals, effect sizes, degrees of freedom and *P* value noted<br>*Give P values as exact values whenever suitable.* |
| ☒ | ☐ | For Bayesian analysis, information on the choice of priors and Markov chain Monte Carlo settings |
| ☒ | ☐ | For hierarchical and complex designs, identification of the appropriate level for tests and full reporting of outcomes |
| ☒ | ☐ | Estimates of effect sizes (e.g. Cohen's *d*, Pearson's *r*), indicating how they were calculated |

*Our web collection on statistics for biologists contains articles on many of the points above.*

## Software and code

Policy information about availability of computer code

| | |
|---|---|
| Data collection | FACS data were collected by BD FACSDiva; chromatography data were collected with BioRad ChromLab; Immunohistochemistry data were collected with Nikon NIS-Elements. |
| Data analysis | FACS data were analyzed by FlowJo 10.5; SPR data were analyzed by BIAevaluate 4.0.1; NMR data were processed using NMRPipe and analyzed with Sparky; structural images were generated using PyMol2.3.4. |

For manuscripts utilizing custom algorithms or software that are central to the research but not yet described in published literature, software must be made available to editors and reviewers. We strongly encourage code deposition in a community repository (e.g. GitHub). See the Nature Research guidelines for submitting code & software for further information.

## Data

Policy information about availability of data

All manuscripts must include a data availability statement. This statement should provide the following information, where applicable:

- Accession codes, unique identifiers, or web links for publicly available datasets
- A list of figures that have associated raw data
- A description of any restrictions on data availability

All data necessary for replication is included above in the Methods section. The wildtype recombinant mCst-3, as well as all mutant forms of mCst-3 described in the manuscript, will be available on request by qualified researchers for their own use.

# Field-specific reporting

Please select the one below that is the best fit for your research. If you are not sure, read the appropriate sections before making your selection.

- [x] Life sciences
- [ ] Behavioural & social sciences
- [ ] Ecological, evolutionary & environmental sciences

For a reference copy of the document with all sections, see nature.com/documents/nr-reporting-summary-flat.pdf

# Life sciences study design

All studies must disclose on these points even when the disclosure is negative.

| | |
|---|---|
| Sample size | The type of experiments we performed are mostly biochemical/biophysical experiments, which as a rule of thumb are much more reproducible than experiments involving live animals. We normally perform these experiments at least three times and found the reproducibility was excellent. |
| Data exclusions | No data were excluded. |
| Replication | We have performed all experiments at least 3 times and found our results were very reproducible. |
| Randomization | This is not relevant to our study as all experiments are very biochemical/biophycial in nature. |
| Blinding | Blinding is not relevant to our study because our study doesn't involve group allocation. |

# Reporting for specific materials, systems and methods

We require information from authors about some types of materials, experimental systems and methods used in many studies. Here, indicate whether each material, system or method listed is relevant to your study. If you are not sure if a list item applies to your research, read the appropriate section before selecting a response.

## Materials & experimental systems

| n/a | Involved in the study |
|---|---|
| [ ] | [x] Antibodies |
| [ ] | [x] Eukaryotic cell lines |
| [x] | [ ] Palaeontology and archaeology |
| [ ] | [x] Animals and other organisms |
| [x] | [ ] Human research participants |
| [x] | [ ] Clinical data |
| [x] | [ ] Dual use research of concern |

## Methods

| n/a | Involved in the study |
|---|---|
| [x] | [ ] ChIP-seq |
| [ ] | [x] Flow cytometry |
| [x] | [ ] MRI-based neuroimaging |

## Antibodies

| | |
|---|---|
| Antibodies used | goat anti-mouse cystatin C (AF1238, R&D systems) |
| Validation | The polyclonal goat anti-mouse Cst-3 antibody was affinity purified using NS0-derived recombinant mouse cst-3. The manufacturer stated that the specificity was evaluated by ELISA and WB and that the antibody was also suitable for IHC. In our hands we found the antibody was indeed highly specific for WB and FACS applications The manufacture website also listed one paper using the antibody for IHC application (PMID: 30760929). |

## Eukaryotic cell lines

Policy information about cell lines

| | |
|---|---|
| Cell line source(s) | CHO-K1 (ATCC), 293-Freestyle (Thermo Fisher) |
| Authentication | CHO-K1 cell was a gift from Dr. Jeffery Esko at UC San Diego and not authentificated in our lab. 293-Freestyle cells were directly purchased from Thermo Fisher. |
| Mycoplasma contamination | CHO-K1 cells were not tested for mycoplasma contamination. 293-F cells were purchased from Thermo Fisher and was tested by manufacture for absence of mycoplasma contamination. |
| Commonly misidentified lines (See ICLAC register) | *Name any commonly misidentified cell lines used in the study and provide a rationale for their use.* |

## Animals and other organisms

Policy information about studies involving animals; ARRIVE guidelines recommended for reporting animal research

| | |
|---|---|
| Laboratory animals | Mice, BL/6, Male, 10-week of age. |
| Wild animals | *Provide details on animals observed in or captured in the field; report species, sex and age where possible. Describe how animals were caught and transported and what happened to captive animals after the study (if killed, explain why and describe method; if released, say where and when) OR state that the study did not involve wild animals.* |
| Field-collected samples | *For laboratory work with field-collected samples, describe all relevant parameters such as housing, maintenance, temperature, photoperiod and end-of-experiment protocol OR state that the study did not involve samples collected from the field.* |
| Ethics oversight | *Identify the organization(s) that approved or provided guidance on the study protocol, OR state that no ethical approval or guidance was required and explain why not.* |

Note that full information on the approval of the study protocol must also be provided in the manuscript.

## Flow Cytometry

### Plots

Confirm that:

- [x] The axis labels state the marker and fluorochrome used (e.g. CD4-FITC).
- [x] The axis scales are clearly visible. Include numbers along axes only for bottom left plot of group (a 'group' is an analysis of identical markers).
- [ ] All plots are contour plots with outliers or pseudocolor plots.
- [x] A numerical value for number of cells or percentage (with statistics) is provided.

### Methodology

| | |
|---|---|
| Sample preparation | CHO-K1 cells were lifted using Accutase from cell culture plates. |
| Instrument | BD LSR Fortessa |
| Software | BD FACSDiva for data collection, FlowJo 10.5 for data analysis |
| Cell population abundance | We use homogeneous CHO-K1 cells in the experiment |
| Gating strategy | All live cells were gated and used in the histogram plots. |

- [ ] Tick this box to confirm that a figure exemplifying the gating strategy is provided in the Supplementary Information.
